# Coexpression patterns define epigenetic regulators associated with neurological dysfunction

**DOI:** 10.1101/gr.239442.118

**Published:** 2019-04

**Authors:** Leandros Boukas, James M. Havrilla, Peter F. Hickey, Aaron R. Quinlan, Hans T. Bjornsson, Kasper D. Hansen

**Affiliations:** 1 Human Genetics Training Program, Johns Hopkins University School of Medicine, Baltimore, Maryland 21205, USA;; 2 McKusick-Nathans Institute of Genetic Medicine, Johns Hopkins University School of Medicine, Baltimore, Maryland 21205, USA;; 3 Department of Human Genetics, University of Utah, Salt Lake City, Utah 84112, USA;; 4 Molecular Medicine Division, The Walter and Eliza Hall Institute of Medical Research, Parkville, Victoria 3052, Australia;; 5 Department of Medical Biology, The University of Melbourne, Parkville, Victoria 3010, Australia;; 6 Department of Biomedical Informatics, University of Utah, Salt Lake City, Utah 84108, USA;; 7 USTAR Center for Genetic Discovery, University of Utah, Salt Lake City, Utah 84108, USA;; 8 Department of Pediatrics, Johns Hopkins University School of Medicine, Baltimore, Maryland 21287, USA;; 9 Faculty of Medicine, University of Iceland, 101 Reykjavík, Iceland;; 10 Landspitali University Hospital, 101 Reykjavík, Iceland;; 11 Department of Biostatistics, Johns Hopkins Bloomberg School of Public Health, Baltimore, Maryland 21205, USA

## Abstract

Coding variants in epigenetic regulators are emerging as causes of neurological dysfunction and cancer. However, a comprehensive effort to identify disease candidates within the human epigenetic machinery (EM) has not been performed; it is unclear whether features exist that distinguish between variation-intolerant and variation-tolerant EM genes, and between EM genes associated with neurological dysfunction versus cancer. Here, we rigorously define 295 genes with a direct role in epigenetic regulation (writers, erasers, remodelers, readers). Systematic exploration of these genes reveals that although individual enzymatic functions are always mutually exclusive, readers often also exhibit enzymatic activity (dual-function EM genes). We find that the majority of EM genes are very intolerant to loss-of-function variation, even when compared to the dosage sensitive transcription factors, and we identify 102 novel EM disease candidates. We show that this variation intolerance is driven by the protein domains encoding the epigenetic function, suggesting that disease is caused by a perturbed chromatin state. We then describe a large subset of EM genes that are coexpressed within multiple tissues. This subset is almost exclusively populated by extremely variation-intolerant genes and shows enrichment for dual-function EM genes. It is also highly enriched for genes associated with neurological dysfunction, even when accounting for dosage sensitivity, but not for cancer-associated EM genes. Finally, we show that regulatory regions near epigenetic regulators are genetically important for common neurological traits. These findings prioritize novel disease candidate EM genes and suggest that this coexpression plays a functional role in normal neurological homeostasis.

The chromatin landscape of any cell is shaped and maintained by the epigenetic machinery (EM), hereafter defined as the group of proteins that can catalyze the addition or removal of epigenetic marks (writer or erasers, respectively), bind to preexisting marks (readers), or use the energy of ATP hydrolysis to alter the local chromatin environment via mechanisms such as nucleosome sliding (remodelers) (Fahrner and Bjornsson 2014; Allis and Jenuwein 2016). Recently, some EM genes have been associated with human diseases, with the most prevalent disease phenotypes falling broadly under the categories of neurological dysfunction (De Rubeis et al. 2014; McCarthy et al. 2014; Bjornsson 2015; Singh et al. 2016; Deciphering Developmental Disorders Study 2017), and cancer (Garraway and Lander 2013; Vogelstein et al. 2013; Feinberg et al. 2016); those associations have indicated that the vast majority of known disease-causing EM genes are haploinsufficient (Garraway and Lander 2013; Bjornsson 2015).

This study addresses three main questions. First, how many additional disease candidate EM genes are there? Existing estimates (Khare et al. 2012; Medvedeva et al. 2015) suggest that EM genes with ascribed roles in disease only form a minority of the whole group. Thus, the number of additional disease candidates that a comprehensive EM gene list will harbor is unclear. It is also unknown whether disease genes tend to be evenly distributed among classes (e.g., erasers versus remodelers) and subclasses (e.g., histone methyltransferases versus histone acetyltransferases) of the machinery; such patterns could reflect the relative contribution of those categories to normal cellular function. Second, is the lost epigenetic function of these genes the most likely cause of disease? Studies in model systems have indicated that the domains mediating the epigenetic function can sometimes be dispensable (Dorighi et al. 2017; Rickels et al. 2017). This raises the possibility that, even among known EM disease genes, the phenotype might have some alternative mechanistic basis. Third, are there expression signatures characteristic of disease candidates? In other words, are the expression patterns of EM genes that are intolerant to variation different from those of variation-tolerant EM genes? Related to this question, it would be of particular interest if there also exist expression signatures that distinguish between EM genes associated with neurological dysfunction versus those associated with cancer. Such signatures could not only prioritize candidate genes for specific phenotypes, but also provide insights into novel disease mechanisms. To answer these questions, we performed a systematic investigation of the human epigenetic machinery with respect to its composition, tolerance to variation, and expression in a diverse set of tissues.

## Results

### The modular composition of the epigenetic machinery

**Figure 1.**
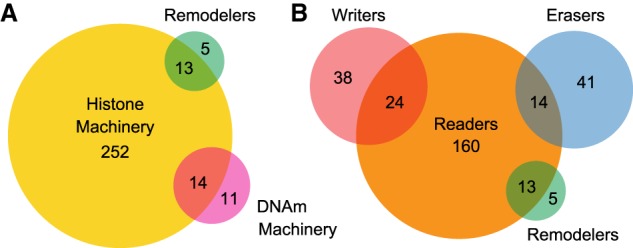
The modular composition of the epigenetic machinery. (*A*) Venn diagram illustrating the three broad categories of the epigenetic machinery (histone machinery, DNA methylation machinery, and remodelers), their relative sizes, and their mutual relationships. (*B*) Venn diagram illustrating the four broad “action” categories of the machinery (writers, erasers, remodelers, and readers), their relative sizes, and their mutual relationships. The modularity of this organization is evident, with some reader components exhibiting enzymatic functions and/or more than one reading function. In contrast, the individual enzymatic component types are pairwise mutually exclusive.

We defined EM genes as genes whose protein products contain domains classifying them as chromatin remodelers, or as writers/erasers/readers of DNA or histone methylation, or histone acetylation. Then, we utilized the UniProt database (The UniProt Consortium 2015), combined with InterPro domain annotations (Hunter et al. 2009), to systematically compile a list of all such human genes (Methods; a full list of the domains used for classification is provided in Supplemental Table S1). This stringent, domain-based definition minimizes the risk of false positives. We found a total of 295 EM genes (Fig. 1A,B; Supplemental Table S2; http://www.epigeneticmachinery.org/), the vast majority of which belong to the histone machinery, and only a small fraction are remodelers or components of the DNA methylation machinery (Fig. 1A). The two latter categories overlap the histone machinery; most remodelers are also readers of either histone methylation or acetylation, whereas the overlap between the DNA methylation and histone components is multifaceted (Supplemental Results).

Considering the categorization of EM genes into readers, writers, erasers and remodelers, we found that the readers make up the biggest group (*n* = 211) and the remodelers the smallest (*n* = 18) (Fig. 1B). The writer and eraser groups are comparable in size (*n* = 62 and *n* = 55, respectively) (Fig. 1B). We observed that the three enzymatic categories (writers, erasers, and remodelers) are pairwise mutually exclusive (Fig. 1B). In contrast, we saw a subgroup of 51 genes encoding proteins that harbor both an enzymatic and a reader domain (Fig. 1B), suggesting that these factors have dual epigenetic function; we will refer to these genes as dual-function EM genes. In general, dual-function writers tend to catalyze the addition of the same mark they read; this is also true for dual-function histone demethylases, the only eraser category with some members that have reading activity. However, there are exceptions: histone methylation readers that enzymatically only function as remodelers (*n* = 11; nine of these are members of the CHD family), or DNA methyltransferases (*n* = 2), and one dual-function histone methyltransferase that only reads DNA methylation. Furthermore, within the reader category, there are 32 genes capable of recognizing more than one type of mark, indicating their participation in crosstalk between different parts of the machinery; we termed those dual readers, and found that some of them (*n* = 7) also have enzymatic activity. Moreover, we observed that the same reading function can be mediated by different domains within a single protein; among the 178 readers of histone methylation we found 23 proteins which contain two distinct reading domains. We also observe that nine of the domains defining EM genes can be present in multiple copies within the same gene, with the exact multiplicity ranging from two to eight (calculated for the EM genes in Supplemental Table S3). Finally, using a previously generated, high-confidence list of 1254 human transcription factors (Vaquerizas et al. 2009; Barrera et al. 2016), we found that 20 EM genes (12 of which are members of the PRDM family of histone methyltransferases) have a DNA-binding domain found in transcription factors, suggesting their involvement in more than one aspect of transcriptional regulation (Supplemental Table S4).

### The human epigenetic machinery is highly intolerant to variation

**Figure 2.**
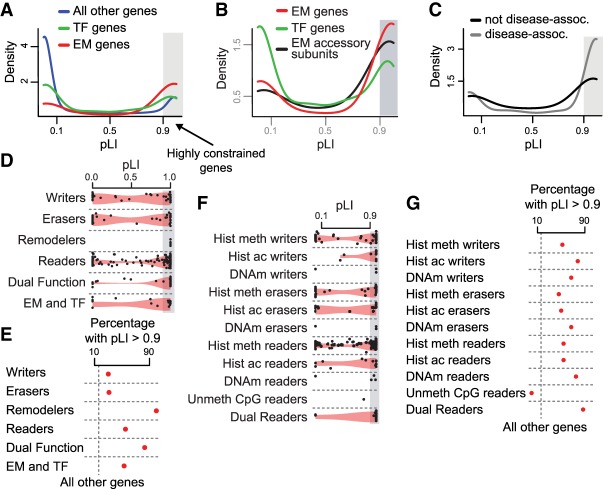
A large subset of epigenetic regulators are very intolerant to variation. (*A*) The pLI distributions of EM genes (red curve), TF genes (green curve), and all other genes (blue curve). (*B*) The pLI distributions of EM genes (red curve), genes encoding for accessory subunits of EM protein complexes (black curve), and TF genes (green curve). (*C*) The pLI distribution of disease-associated EM genes versus non-disease–associated EM genes. (*D–G*) The pLI distributions (*D*,*F*) and percentage of genes with pLI > 0.9 (*E*,*G*) of individual classes of EM genes. The shaded gray area (*A–D*,*F*) indicates highly constrained genes (>0.9). The vertical dashed gray line (*E*,*G*) corresponds to the percentage of all other genes with pLI > 0.9.

To identify novel EM disease candidates, we systematically investigated the tolerance of the entire EM group to loss-of-function variation. To achieve this, we used the ExAC database coupled with the pLI score (Lek et al. 2016), a metric which ranges between 0 and 1 and measures the extent to which a given gene tolerates heterozygous loss-of-function variants. In particular, genes with a pLI of more than 0.9 have been described as highly dosage sensitive (Lek et al. 2016), with virtually all known haploinsufficient human genes belonging to this category (Lek et al. 2016). A similar approach, focused only on the histone methylation machinery, was very recently used to derive candidate genes for developmental disorders (Faundes et al. 2018). In total, ExAC provides a pLI for 18,225 human genes, of which 281 are EM genes. First, we observed that EM genes have significantly higher pLI scores compared to all other genes (Wilcoxon rank-sum test, *P* < 2.2 × 10^−16^) (Fig. 2A) and show substantial enrichment in the highly intolerant category (Fisher's exact test, *P* < 2.2 × 10^−16^, odds ratio = 7.7). We note that there are many EM genes with a pLI score between 0.7 and 0.9, a range which is almost absent for other genes. Given that pLI is a measure of haploinsufficiency, genes encoded on the X and Y Chromosomes were not considered in this comparison (for details on EM genes encoded on the sex chromosomes, see Supplemental Results).

We next compared EM to TF genes; this is a natural comparison, because EM proteins are usually recruited to target sites by TFs (Lappalainen and Greally 2017) and TF genes have previously been shown to be mostly haploinsufficient (Jimenez-Sanchez et al. 2001; Seidman and Seidman 2002). Using the 1155 TF genes in ExAC, we first showed that they have significantly higher pLI compared to other genes (Wilcoxon rank-sum test, *P* < 2.2 × 10^−16^) (Fig. 2A), although they are less dosage sensitive than previously suggested (Jimenez-Sanchez et al. 2001), illustrating the value of our comprehensive approach. Comparing TF to EM genes, however, we observed that EM genes have higher pLIs (Wilcoxon rank-sum test, *P* < 2.2 × 10^−16^) (Fig. 2A) and are more strongly enriched in the highly dosage sensitive category (Fisher's exact test, *P* < 2.2 × 10^−16^, odds ratio = 4.4).

Because it is known that many EM gene products function as parts of multi-subunit complexes (Carrozza et al. 2003; Clapier and Cairns 2009; Laugesen and Helin 2014; Rao and Dou 2015), we reasoned that genes encoding for accessory subunits of these complexes (which are not categorized as EM genes by our definition), would also show a similar intolerance to variation. We thus assembled a list of 95 non-EM accessory subunit genes and 46 EM subunit genes, spanning a total of 19 complexes with chromatin modifying activities (Methods; Supplemental Table S5). As expected, the 46 EM subunit genes are very dosage sensitive; 80% of these genes have a pLI > 0.9. Considering the accessory subunits, their pLI distribution confirmed that they are more constrained than all other genes, as well as TF genes; however, they are slightly less constrained than all EM genes (Fig. 2B). A more detailed investigation revealed that in general, each complex contains multiple accessory and EM subunits that are highly constrained (Supplemental Fig. S1). Specifically, across all 19 complexes, the median percentage of accessory and EM subunits with a pLI > 0.9 was 64% and 100%, respectively.

### Identification of new disease candidate epigenetic regulators

After splitting EM genes into those with existing disease associations and those with no reported link to disease (the latter constituting ∼70%) (Methods), we discovered that in both the disease- and the non-disease-associated groups there exist many EM genes with elevated pLI scores (Fig. 2C), although the disease-associated ones exhibit higher skewing. It is notable that EM genes which are only associated with cancer have high pLIs (median pLI = 0.98, percentage with pLI > 0.9 = 65%). There is no a priori reason to expect this for somatic cancer driver genes, because pLI scores were derived after only excluding individuals with severe pediatric disease (Lek et al. 2016). Overall, this result suggests the existence of additional EM disease genes. Among 162 EM genes in ExAC with no reported link to disease, 78 have a pLI greater than 0.9 (percentage with pLI > 0.9 = 78/162 = 48%). Additionally, there are 24 EM genes that have only been associated with cancer but whose pLI is greater than 0.9, suggesting that they also cause some other disease phenotype. In total, this leads to 102 novel EM disease candidates (Supplemental Table S6). Finally, the same approach for the EM accessory subunits (Methods) highlighted 39 new disease candidate genes whose phenotypic consequences are likely to arise through similar mechanisms as in the case of EM genes (Supplemental Table S7).

### Dual-function epigenetic regulators and remodelers are the most variation-intolerant categories

We next explored the loss-of-function variation tolerance of the different types of machinery components. Chromatin remodelers are an extremely intolerant group, whereas both the writers and the erasers are equally distributed among the high and low pLI groups (Fig. 2D,E). Collectively, the three enzymatic EM classes show high mutational constraint (Fisher's exact test, *P* = 9.82 × 10^−9^, odds ratio = 4.1 for enrichment of EM genes with enzymatic but not reading function in the pLI > 0.9 category). This is unusual since enzymes are in general haplosufficient (Jimenez-Sanchez et al. 2001). Readers are more skewed toward the high pLI category than writers and erasers (Fig. 2D,E). The 20 EM genes that also contain TF DNA-binding domains show a pLI distribution which mirrors that of the whole EM group (Fig. 2D,E). Despite the differences between the single-function classes, dual-function EM genes are extremely constrained, regardless of the specific enzymatic function; this underscores the importance of this unique category (Fig. 2D,E). Consistent with this, a more detailed breakdown into EM categories (Fig. 2F,G; Supplemental Results) highlighted dual readers as a very intolerant group (Fig. 2F,G). Finally, we found that multiple histone modifiers with seemingly redundant biochemical activities (same amino acid substrate specificity) (Methods; Supplemental Table S8) can be highly constrained (Supplemental Results; Supplemental Fig. S2), as also observed for DNA methylation writers/erasers (Fig. 2F).

### The intolerance to variation is primarily driven by the domains mediating the epigenetic function

A recent study showed that *Drosophila* embryos with a catalytically inactive version of Trr (a homolog of the mammalian histone methyltransferases KMT2C and KMT2D) develop normally, despite altered histone methylation patterns (Rickels et al. 2017). This example shows that in some cases the inactivation of an epigenetic domain (in this case, the SET domain) might not have severe, easily detectable consequences. Our previous analysis is unable to determine if the observed variation intolerance is driven by the presence of epigenetic, or other nonepigenetic domains. We therefore asked if the EM-specific domain(s) in an EM gene had a different local mutational constraint than other domains in the same gene. To answer this, we used the constrained coding region (CCR) model (Havrilla et al. 2019) to examine the mutational constraint of EM genes at the domain level. Specifically, we classified a given domain as constrained or not; this classification reflects how devoid a domain is of missense or loss-of-function mutations in the gnomAD database (Lek et al. 2016) compared to other similar regions (Methods). We were able to study 237 out of 295 EM genes.

**Figure 3.**
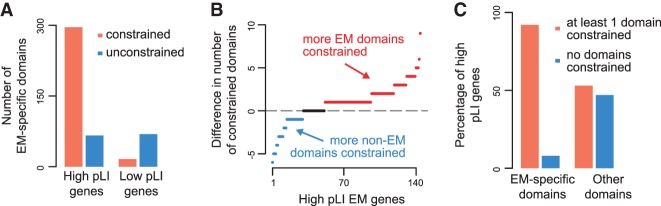
The protein domains known to mediate epigenetic functions drive the observed constraint of EM genes. (*A*) The number of constrained and unconstrained EM-specific protein domains of high pLI (>0.9) EM genes versus low pLI (<0.1) EM genes. (*B*) The within-gene differences in the total number of EM-specific constrained domains versus other constrained domains. Each dot corresponds to a gene. Red dots indicate genes with more EM-specific constrained domains; blue dots indicate genes with more other constrained domains; black dots indicate genes with an equal number of constrained EM-specific and other domains. (*C*) The percentage of high pLI EM genes with at least one constrained EM-specific domain versus the corresponding percentage with at least one constrained other domain.

Under the hypothesis that EM-specific domains are not contributing to the observed variation intolerance of EM genes, there should be no difference in the constraint of EM-specific domains found in high pLI versus low pLI EM genes. In contrast to this, we found that, collectively, the EM-specific domains of high pLI genes (greater than 0.9 pLI) are much more likely to be constrained than those of low pLI genes (less than 0.1 pLI; 82% versus 19%) (Fig. 3A). To explore this further, we restricted our analysis to high pLI genes, and compared the contribution of EM-specific domains to that of other domains. First, at the individual gene level, we found that for most EM genes (65%), the number of EM-specific constrained domains exceeds that of other constrained domains (Fig. 3B). Furthermore, almost all high pLI EM genes (92%) have at least one constrained EM-specific domain, whereas approximately half (47%) have no other constrained domains (Fig. 3C). In fact, there are 54 high pLI EM genes that do not contain other domains. Notable exceptions in this analysis are four high pLI members of the PRDM family, for which the C2H2-like zinc fingers are the main drivers of variation intolerance (Supplemental Fig. S3).

We note that our approach is overall conservative, as there are domains without catalytic or reading activity (which are thus labeled as non-EM-specific), that are nevertheless found only in EM genes (Methods). Returning to our initial example, in *KMT2D* we see that the catalytic SET domain is constrained, as is its associated post-SET domain, and five of the seven PHD-fingers.

Finally, we repeated this analysis using a quantitative version of domain-specific constraint, in which each domain is assigned a score from 0 to 100 (with greater values indicating more constrained domains) (Methods). The results we obtained regarding the relative constraint of EM-specific versus other domains recapitulated the findings described above (Supplemental Fig. S4A,B). Additionally, this revealed that multiple identical copies of a domain within a single gene can differ with respect to their constraint (Supplemental Figs. S4C, S5). This could reflect different contributions of these identical copies to gene function, although it is possible that this variability in domain constraint is a consequence of inadequate sampling of variation (since it has been estimated that even with 500,000 individuals, ∼10% of protein-coding variation will be captured) (Zou et al. 2016; Havrilla et al. 2019).

### A large subset of the epigenetic machinery is coexpressed

To identify functional properties specific to variation-intolerant EM genes, we systematically explored the expression patterns of the whole group across a spectrum of adult tissues, using publicly available RNA-seq data (The GTEx Consortium 2015). We selected 28 tissues on the basis of sample size and diversity in physiological function (Methods; Supplemental Table S9). First, we discovered that virtually all EM genes are expressed in a non-tissue-specific manner, similarly to what is observed for known housekeeping genes (Supplemental Results; Supplemental Methods; Supplemental Fig. S6A,B; Supplemental Table S10), with the exception of a small number that showed testis-specific expression (Supplemental Fig. S6C). Hence, tissue specificity cannot account for the differences in variation tolerance within the EM group; we also found that it cannot explain the high mutational constraint of EM genes versus TF genes and other genes, after restricting the pLI comparison to very broadly expressed genes from both groups (Supplemental Results; Supplemental Fig. S7). Similarly, we saw that the absolute expression level does not robustly discriminate between EM genes with high pLI (>0.9) and the rest of the machinery (Supplemental Fig. S8).

However, although EM genes show ubiquitous expression, within any given tissue there is inter-individual variability in their expression levels (Supplemental Fig. S9). We noticed that in several cases, EM genes show coordinated fluctuations in their expression levels across individuals (Supplemental Fig. S9A). We reasoned that this might reflect the precise epigenetic regulation of the transcriptional programs operating within each cell. Therefore, we hypothesized that EM genes whose expression patterns display this coordinated behavior (coexpression) would differ in their mutational constraint from those who do not. To test this, we constructed tissue-specific coexpression networks and determined modules of coexpressed genes using Weighted Correlation Network Analysis (WGCNA) (Zhang and Horvath 2005; Methods).

**Figure 4.**
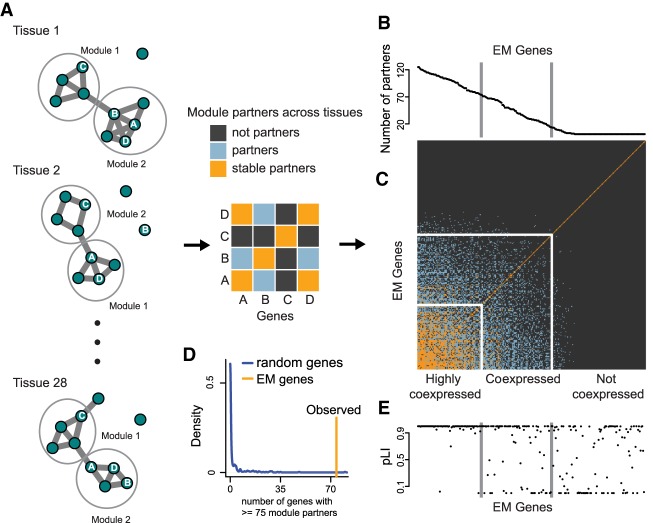
A large subset of the components of the epigenetic machinery exhibit unusually high levels of coexpression. (*A*) Schematic illustrating our definition and identification of module partners. WGCNA was used to construct tissue-specific coexpression networks and modules for 28 tissues profiled in GTEx. We determined if two EM genes were module partners (part of the same module in 10–14 tissues) or stable module partners (part of the same module in >14 tissues). (*B,C*) The number of module partners for each EM gene and the module partner matrix, where rows and columns are ordered as in *B*. We define three groups of EM genes—highly coexpressed, coexpressed, and not coexpressed—based on their number of module partners. (*D*) The pLI for each EM gene, ordered by its number of module partners as in *B*. (*E*) The size of the (highly) coexpressed group of EM genes compared to 300 draws of 270 random genes, in which the random genes are selected to have a similar expression level across tissues compared to EM genes (Supplemental Fig. S10).

We noticed that for all tissues, EM genes were grouped in a few large modules (median two modules across tissues, range 0–4), with a substantial number of genes not belonging to any module (singletons; median 106 singletons across tissues, range 9–270). We asked if the division of EM genes into genes belonging to large modules and genes being singletons was stable across tissues. Because these modules were estimated separately for each tissue, it is not obvious how to compare them across tissues, and modules are affected by noise resulting from differences in sample size, and other sources. To perform the comparison, we defined two genes to be module partners if they belonged to the same module in at least 10 tissues, stable module partners if they belonged to the same module in at least 14 tissues, and not module partners if they belonged to the same module in less than 10 tissues (light blue, orange, and dark gray squares, respectively, in cartoon Fig. 4A).

For each gene, we computed the number of module partners and then ordered EM genes according to this score (Fig. 4B). We next collectively visualized the pairwise partnership statuses among EM genes in a symmetric matrix, keeping this ordering for both rows and columns (Fig. 4C, blue). We observed a distinct clustering, with a set of genes that are predominately stable module partners with each other (Fig. 4C, orange), a large set of genes with no module partners (Fig. 4C, dark gray), and a transition between these two groups (Fig. 4C). We noted that the transition occurs as the number of module partners increases, meaning that EM genes not only have more partners, but they are also stable partners with the majority of them.

We then divided EM genes into three groups: (1) a group of 74 genes with at least 75 module partners; we call this group of EM genes “highly coexpressed”; (2) a group of 83 genes with between 15 and 74 module partners; we call this group “coexpressed”; and (3) a group of 113 genes with fewer than 15 module partners; we call this group “not coexpressed.” To assess the statistical significance of the size of these groups, we compared our results to those obtained with randomly chosen genes (Supplemental Fig. S10) and found that the groups of highly coexpressed as well as coexpressed EM genes are much larger than expected by chance (Fig. 4D; Supplemental Fig. S11A,B). We also established that our results are robust to the choice of cutoffs, the presence of sample outliers, and the exact network reconstruction method used (Methods; Supplemental Fig. S11C, Supplemental Fig. S12). Finally, we note that our across-tissue coexpression analysis provides confidence that our findings are not driven by the cell-type heterogeneity present in these tissue samples.

### Dual-function epigenetic regulators are enriched in the highly coexpressed group and are coexpressed with multiple other categories

**Figure 5.**
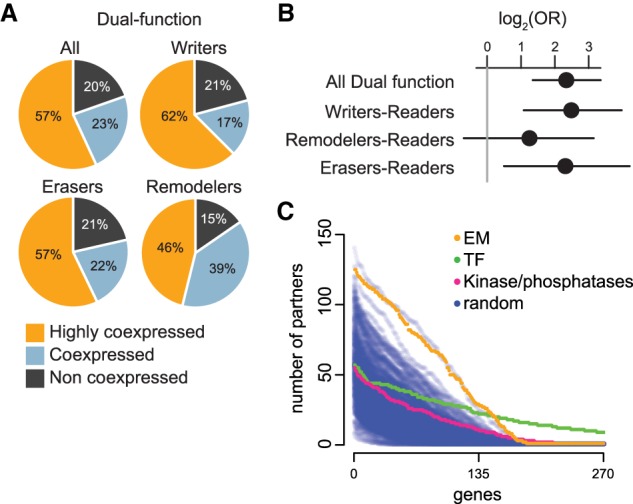
Dual-function EM genes are enriched within the highly coexpressed group. (*A*) The distribution of dual-function EM genes (collectively and separately for each enzymatic group) within the three coexpression categories. (*B*) Log odds ratios and 95% confidence intervals for enrichment of dual-function EM genes (collectively and separately for each enzymatic group) in the highly coexpressed category. The vertical gray line at 0 corresponds to statistical significance. (*C*) Blue dots correspond to randomly chosen genes, sampled in sets of 270 genes from genes with a median expression (log(RPKM + 1)) greater than 0.5 in at least half the tissues, to match the expression of EM genes (as in Fig. 4D). Orange, green, and pink dots correspond to EM genes, TF genes, and protein kinases/phosphatases, respectively. Each dot corresponds to a single gene, and its position along the *y*-axis corresponds to the number of other genes with which it partners. The genes are ordered on the *x*-axis according to the number of their partners. This figure also serves as a sensitivity analysis with respect to the number of partners for this particular tissue cutoff.

To better understand the coexpression phenomenon, we examined whether some EM categories are overrepresented within the highly coexpressed group. We first observed an enrichment for dual-function EM genes (Fig. 5A,B). This was driven by the enrichment of dual-function writers, as well as dual-function erasers (Fig. 5A,B). Although we found six highly coexpressed dual-function remodelers, there was no statistically significant overrepresentation compared to the coexpressed and not coexpressed groups (Fig. 5A,B). We then performed a breakdown of the partners of highly coexpressed dual-function histone methyltransferases and acetyltransferases. We observe that both of these EM groups partner with their corresponding readers and erasers, as well as with remodelers (Supplemental Fig. S13). In addition, the two groups partner with each other and with the DNA methylation machinery (Supplemental Fig. S13). This is partly expected given the large number of partners of highly coexpressed genes (more than 75 per definition) compared to the size of the individual EM categories.

We next wanted to investigate if the observed coexpression is related to the involvement of EM genes in transcriptional regulation, their organization into writers/erasers/readers, or both. To test the first possibility, we used TF genes as a reference group, whereas to test the second possibility we used genes encoding for protein phosphorylation writers/erasers (i.e., kinases/phosphatases) (Manning et al. 2002; Chen et al. 2017). We discovered that neither of these two classes of genes are coexpressed (Fig. 5C; Supplemental Fig. S14), suggesting that the coexpression is a unique property of EM genes likely reflecting both their role in transcription and their modular composition.

### The highly coexpressed epigenetic regulators are extremely intolerant to variation and enriched for genes causing neurological dysfunction

**Figure 6.**
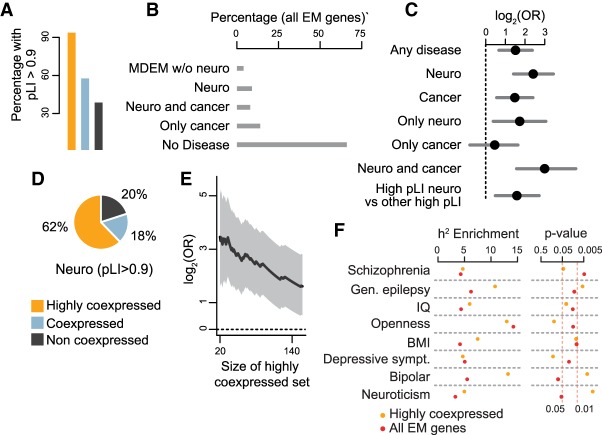
EM genes linked to disorders with neurological dysfunction demonstrate significant enrichment within the highly coexpressed category. (*A*) The percentage of EM genes with pLI > 0.9 in each of the coexpression categories. (*B*) The percentage of EM genes that are associated with different types of disease; individual disease categories are mutually exclusive. (MDEM) Mendelian disorders of the epigenetic machinery; (Neuro) includes autism, schizophrenia, developmental disorders, and MDEM whose phenotype includes dysfunction of the central nervous system (Methods). (*C*) Log odds ratios and 95% confidence intervals for enrichment of different subsets of EM genes in the highly coexpressed category. The dashed vertical line at 0 corresponds to statistical significance. (*D*) The percentage of EM genes that are associated with neurological dysfunction and have pLI > 0.9 in each of the coexpression categories. (*E*) Odds ratio (black line) and 95% confidence interval (shaded area) for enrichment of EM genes associated with neurological dysfunction in the highly coexpressed group, as a function of the size of the highly coexpressed group. For all sizes, the comparison was performed against the not coexpressed group. (*F*) Estimates for enrichment of explained heritability, and unadjusted *P*-values, for eight traits and two sets of regulatory features: regions marked by H3K27ac in brain within 1 Mb of the transcription start site of all-EM (red dots) or highly coexpressed (orange dots) EM genes.

If this coexpression of EM genes is functionally important, we would anticipate a relationship with their mutational constraint. Indeed, examination of the pLI scores of the three coexpression groups revealed a very clear association (Fig. 4E), with almost all highly coexpressed genes being extremely intolerant to variation (percentage of genes with pLI > 0.9 is >90%), coexpressed genes exhibiting intermediate intolerance, and the not coexpressed group being the least intolerant (Fig. 6A).

We next asked whether, in addition to being very constrained, coexpressed EM genes are also preferentially associated with specific disease phenotypes. To perform this analysis, we first used our full list of EM genes to obtain a comprehensive picture of those links to disease. We examined associations with Mendelian disorders, cancer, and complex disorders (Fig. 6B; Methods; Supplemental Results). EM genes associated with any one of those disease phenotypes were enriched within the highly coexpressed group (Fig. 6C). We thus sought to examine whether this enrichment was driven by associations with particular disease categories.

We found a marked enrichment for genes causing neurological dysfunction (Fig. 6C); genes implicated in cancer were also enriched, although less (Fig. 6C). We next tried to disentangle the contributions stemming from the associations with cancer versus neurological dysfunction. To accomplish this, we partitioned the EM genes into genes only associated with neurological dysfunction, genes only associated with cancer, and genes associated with both. Genes only associated with neurological dysfunction were still enriched, whereas we did not observe significant enrichment for genes only associated with cancer (Fig. 6C). Subsequently, we asked if this result was a consequence of the association between coexpression and pLI and restricted the analysis to EM genes with high pLI (>0.9). We found that these associated with neurological dysfunction were still significantly enriched in the highly coexpressed category (Fig. 6C,D). As expected given the overrepresentation of dual-function genes (which are themselves enriched in the highly coexpressed subset) in the set of genes associated with both neurological dysfunction and cancer, the latter were particularly enriched in the highly coexpressed group (Fig. 6C). We then examined the impact of the definition of the highly coexpressed group on the strength of the enrichment by varying the coexpression cutoff, while using the constant set of non-coexpressed EM genes as a control. As expected, this showed that the enrichment increased as the stringency of our cutoff increased (Fig. 6E).

### Brain-specific regulatory elements of highly coexpressed epigenetic regulators are enriched for SNPs that explain the heritability of common neurological traits

The preceding results establish that rare, coding variants in highly coexpressed EM genes preferentially cause neurological dysfunction. We next asked whether common variation in regulatory regions surrounding these genes, as well as all EM genes collectively, contributes to neurological disease risk. To achieve this, we used a set of brain enhancers (defined by the presence of H3K27 in one or more of 87 distinct brain regions) (Vermunt et al. 2014; Methods) and labeled every such enhancer within 1 Mb of the TSS of EM genes as an EM-regulatory region. We then performed stratified LD score regression (Finucane et al. 2015) to assess whether these EM-regulatory regions show enrichment for explained heritability in 24 neurological diseases/traits (Methods; Supplemental Fig. S15; Supplemental Table S11) compared to what is expected given the size of the regions and their overlap with regions of known genetic importance.

For seven of the 24 neurological traits, either the highly coexpressed, or the all-EM regulatory regions showed significantly enriched heritability at *P* = 0.05 (corrected for multiple testing within each trait) (Fig. 6F). Two of the seven traits (neuroticism and bipolar disorder) were only significant for the set of highly coexpressed regulatory regions, and two other traits (openness and depressive symptoms) were only significant for the all-EM regulatory regions. The remaining three traits (schizophrenia, general epilepsy, IQ) are significant for both sets of regulatory regions. However, the enrichment of heritability for the highly coexpressed regulatory regions was either exceeding or on par with the enrichment for the all-EM regulatory regions, despite the fact that the former are considerably smaller (15 Mb versus 46 Mb). As a negative control, we also examined five non-neurological traits (Supplemental Fig. S15; Supplemental Table S11). For four of them, we observed that neither set of regions showed heritability enrichment, as expected. The only exception was BMI, in concordance with recent results implicating brain regulatory elements in BMI heritability (Finucane et al. 2015).

### The promoters of highly coexpressed genes of the epigenetic machinery are bound by common *trans*-acting factors

To gain insights into the mechanistic basis of the observed coexpression, we investigated (1) whether these genes are colocalized in the genome, and (2) whether there is evidence that they are regulated by common *trans*-acting factors. It has been observed that highly expressed genes tend to reside in chromosomal clusters in the human genome (Caron et al. 2001), and clustered genes are often coexpressed (Cohen et al. 2000). However, we did not observe any evidence of spatial clustering of EM genes (Supplemental Results; Supplemental Fig. S16).

To test whether shared regulation potentially contributes to coexpression, we asked if the promoters of highly coexpressed EM genes are bound by common *trans*-acting factors. To answer this, we used ENCODE ChIP-seq data from K562 cells (The ENCODE Project Consortium 2012). We chose this cell line because it contains by far the most extensive collection of ChIP-seq data on such factors, and because our coexpression analysis suggests that the coexpression is tissue-independent. We tested each of the 330 factors available from ENCODE for enriched binding at the promoters of the highly coexpressed EM genes relative to those of the non-coexpressed EM genes. Although these factors are a relatively small subset of the 1254 TFs encoded in the human genome (Vaquerizas et al. 2009; Barrera et al. 2016), we found that 53 factors exhibit at least twofold enrichment, in contrast to what is observed for randomly chosen genes, or after permuting the labels of EM genes (Methods; Supplemental Fig. S17) (*P* = 0.02 and *P* = 5 × 10^−4^, respectively). We note that the direction of effect is consistent with our hypothesis: There is only one factor enriched in the non-coexpressed group compared to the highly coexpressed group.

## Discussion

We have performed a systematic investigation of all human genes encoding for epigenetic writers/erasers/remodelers/readers (EM genes). This enables us to make three basic contributions. First, we identify 102 novel disease candidates within this class of genes. Second, we provide evidence that genetic disruption of the epigenetic domains of EM genes is the most likely cause of the disease phenotypes. This suggests that these phenotypes are caused by an abnormal epigenomic state. Third, we discover that coexpression distinguishes a large subset of EM genes that are both extremely variation-intolerant and, independently, enriched for genes causing neurological dysfunction.

We note that our pLI-based approach for disease gene identification, while unbiased, cannot discriminate between genes that cause severe pediatric disease versus genes that lead to lethality at the zygotic or embryonic stage. Additionally, although our local mutational constraint analysis argues that the enzymatic/reading functions are the primary drivers of this intolerance to variation, some EM proteins may participate in biochemical events that affect other, non-chromatin-related cellular functions (Spange et al. 2009; Biggar and Li 2015). The importance of nonhistone protein methylation and acetylation for signal transduction pathways and other molecular activities is not well understood, although there are examples of established functional relevance. These include cases of Cornelia de Lange syndrome caused by defective deacetylation of SMC3, a subunit of the cohesin complex (Deardorff et al. 2012), as well as the regulation of the tumor-suppressor functions of TP53 through acetylation mediated by CBP/EP300 (Liu et al. 1999; Iyer et al. 2004). Further elucidation of such mechanisms will undoubtedly yield more insights into this issue.

Our most unexpected finding is that, among these 295 EM genes, we detected a subset of 74 that are highly coexpressed within tissues, as well as 83 others with an intermediate level of coexpression. The sizes of the two groups and the exact cutoff separating them might be refined with future interrogation of more tissues/cell types, and increases in sample size, but we anticipate the rank ordering of EM genes with respect to their partners to remain accurate. This coexpression appears to unite three seemingly independent properties of the machinery: variation intolerance, association with neurological dysfunction (even after conditioning on haploinsufficiency), and dual-function (enzymatic activity combined with reading function). From a functional standpoint, the clear relationship between coexpression and mutational constraint indicates that the former potentially plays a role in homeostasis and disease predilection. It also suggests a basis for the observed dosage sensitivity, a counterintuitive result given that many EM genes are enzymes and enzymes are usually haplosufficient (Jimenez-Sanchez et al. 2001). For coexpressed enzymes, however, a reduction of the normal amount of protein product present would not be tolerated, since it would compromise the coordinated expression of the module. Given the strong signal for enrichment of genes causing neurological dysfunction, it becomes tempting to speculate that the coexpression might be especially relevant to brain development and function; future examination of EM gene expression during fetal and early childhood development will likely yield profound insights into this. It will also be important to develop methods for experimental perturbations of this coexpression, to help define the precise cellular consequences of its disruption. Finally, prioritization of highly coexpressed EM genes might not only aid in the discovery of new pathogenic variants disrupting the epigenetic machinery, but also provide a starting point for the interpretation of the functional consequences of those variants, particularly in the context of neurological dysfunction.

Most of our work has focused on the disease causing potential of rare coding variation in EM genes, but we have also established that brain-specific regulatory regions surrounding them show enriched heritability signal for multiple common neurological traits. It is noteworthy that these traits include a measure of intellect (IQ) and a seizure phenotype (generalized epilepsy), given that intellectual disability and seizures are among the most common neurological manifestations of the Mendelian disorders of the epigenetic machinery (Bjornsson 2015). For another epileptic phenotype, focal epilepsy, we did not find heritability enrichment for either set of EM genes. However, this GWAS included mostly adult individuals (International League Against Epilepsy Consortium on Complex Epilepsies 2014), and thus probably contains a different genetic signal. We hypothesize that, as large high-quality data sets accumulate, many common traits with a neurodevelopmental origin will be genetically influenced by EM genes.

With respect to the underlying mechanistic basis of the coexpression, one way that such coregulation could be achieved is with shared upstream regulators. Our data on *trans*-acting factor binding at the promoters of EM genes support this possibility. However, a definitive answer to this will only be provided after further delineation of human regulatory circuits, with mapping of enhancer–promoter interactions in different cell types. Currently available data also argue against the formation of multi-subunit complexes between the coexpressed EM gene products (Supplemental Results). Hence, it is possible that the need for coexpression arises not to regulate protein–protein interactions, but because imbalance of the epigenetic system could over time lead to major changes in open versus closed chromatin (Fahrner and Bjornsson 2014).

In summary, our data provide the first evidence of widespread coexpression of epigenetic regulators and link this phenomenon to both variation intolerance and neurological dysfunction, thus opening a new avenue to better understand the role of the human epigenetic machinery in health and disease.

## Methods

### The creation of an epigenetic regulator list

We used InterPro domain annotations as provided by the UniProt database (Hunter et al. 2009; The UniProt Consortium 2015), accessed in June 2016, to generate a list of proteins with at least one domain that classifies them as writers or erasers of histone lysine methylation (Dillon et al. 2005; Shi 2007), writers or erasers of histone lysine acetylation (Marmorstein and Zhou 2014; Seto and Yoshida 2014), readers of the two aforementioned histone modifications (Musselman et al. 2012), and readers of methylated and unmethylated CpG dinucleotides (Lee et al. 2001; Jørgensen and Bird 2002). A full list of all the domains used along with the corresponding InterPro IDs is provided in Supplemental Table S1. Additionally, we included the known human DNA methyltransferases and demethylases (Weissman et al. 2014), as well as the catalytic subunits of the known human chromatin remodeling complexes (Clapier and Cairns 2009). We only included UniProt entries that have been manually annotated and reviewed by the database curators. The full list of all EM genes used in our analyses along with several of their features, is provided in Supplemental Table S2. We then made minor refinements to our list, following a literature search (Supplemental Methods).

Although our categorization of EM-specific domains only includes domains which have some catalytic or reading function, there are domains which were not labeled as EM-specific, but are exclusively or almost exclusively found in EM genes. Two such examples are the pre-SET domain (present in seven proteins, all of which are HMTs) and the post-SET domain (present in 16 proteins, 15 of those are HMTs and the remaining protein has eight domains, all of which are post-SET domains).

To define EM protein complexes, we performed a manual literature curation (Carrozza et al. 2003; Clapier and Cairns 2009; Laugesen and Helin 2014; Rao and Dou 2015), and subsequently assembled a catalog of the EM and accessory subunits of 19 complexes with chromatin modifying activities (Supplemental Table S5).

To group EM histone modifiers according to their amino acid substrate specificities, we classified EM genes as H3K4, H3K27, H3K36, H3K9 methylation writers/erasers (Zhao and Garcia 2015; Hyun et al. 2017), H3K27 acetylation writers (Raisner et al. 2018), and H3K9 acetylation erasers (Supplemental Table S8; Seto and Yoshida 2014).

### Epigenetic regulators with disease associations

For Mendelian disease associations, we included disorders with a phenotype mapping key equal to 3 (indicating sufficient evidence to ascribe causality for a particular gene) in OMIM (https://omim.org/), as accessed in June 2016. We determined which of those syndromes involved dysfunction of the central nervous system based on the corresponding clinical synopses in OMIM. In addition to OMIM, we included genes for which there is strong evidence associating pathogenic protein-coding variants to autism (De Rubeis et al. 2014), schizophrenia (McCarthy et al. 2014; Singh et al. 2016), or developmental disorders (Supplemental Methods; Zech et al. 2016; Deciphering Developmental Disorders Study 2017). In total, this yielded 50 EM genes associated with disorders exhibiting symptoms of abnormal brain function: Eight are linked to autism, schizophrenia, and developmental disorders, and 42 are known to cause a monogenic disorder in OMIM. As can be seen under the “Clinical Synopsis” in OMIM, in each of these disorders the affected children can have a variety of manifestations under the “Neurologic” category. These include intellectual disability of variable severity, seizures, speech delay, apraxia, balance/gait abnormalities, memory defects, and others. Additionally, patients with autism and schizophrenia also exhibit several different symptoms attributable to central nervous system dysfunction (such as seizures and memory deficits). Hence, we concluded that the most clinically meaningful classification of EM genes linked to such diseases is as “associated with neurological dysfunction.”

Recently, Faundes et al. (2018) identified disease candidates within the histone methylation machinery; we excluded these EM genes from our list of novel disease candidates provided in Supplemental Table S6.

Regarding associations with cancer, we first identified EM genes potentially functioning as cancer drivers using: (1) a list of 260 significantly mutated cancer genes, derived from data spanning 21 tumor types (Lawrence et al. 2014), and (2) genes that were predicted to be drivers by at least one of the top three performing methods in Tokheim et al. (2016). Both these studies evaluated genes based on point mutations and small insertions/deletions. We then also included other EM genes that have been reported to be involved in cancer, harboring either point mutations/small indels or structural rearrangements (Shen and Laird 2013; Feinberg et al. 2016). Taken together, the preceding studies show that EM genes are associated with a wide variety of tumor types, both solid and hematological (e.g., renal cell carcinoma, colorectal cancer, lung cancer, melanoma, pancreatic neuroendocrine tumors, T/B cell lymphoma, acute lymphoblastic leukemia, and others). This indicates that they broadly promote tumorigenesis when mutated in somatic cells, in a tissue-independent manner. Therefore, we collectively refer to all these EM genes as “cancer-associated.”

All the aforementioned disease associations are provided in Supplemental Table S2.

### Variation tolerance analysis

pLI scores for heterozygous loss-of-function constraint were downloaded from the ExAC database (Lek et al. 2016). When comparing the pLI distributions of different classes of genes, we excluded genes encoded on the X and Y Chromosomes.

### CCR local constraint score

The CCR model (Havrilla et al. 2019) identifies regions of the genome without any missense or loss-of-function mutations in gnomAD (Lek et al. 2016). Each region devoid of mutations is assigned a CCR percentile score; the greater the difference between the observed and expected coverage-weighted length for regions with similar CpG density, the higher the constraint. As a result, the CCR model extends single gene-wide estimates of constraint to identify subregions within genes that exhibit “local constraint.” We mapped each protein domain to the genome, using the Pbase package (available from Bioconductor at https://bioconductor.org/packages/Pbase) (see also Supplemental Methods). For each EM gene, we restricted our analysis to the single specific isoform for which ExAC provides a pLI score. We then classified a domain as constrained if at least 10% of bases in that domain resided in a genomic region with a CCR percentile score above 90, using Supplemental Table S12. Our rationale for choosing this cutoff was that (1) it had to be a sizable percentage, and (2) in high pLI genes that only contain a single domain (such as *TET3*), that domain had to be constrained. However, to examine whether our results are dependent on the choice of cutoff, we also performed our analysis with a quantitative version of this domain-specific constraint. Specifically, we defined the “CCR local constraint” of a domain to be the percentage of bases in the domain residing in a genomic region with a CCR percentile score above 90. Our analysis yielded results that mirrored those obtained with the binary version of the CCR constraint (Supplemental Fig. S4). The CCR local constraint score (quantitative version) for the protein domains in EM genes is included in Supplemental Table S3.

### GTEx data

RNA-seq data from 28 tissues (Supplemental Table S9) from 449 individuals were downloaded from the GTEx portal, release V6p. Those 28 tissues were selected based on differences in physiological function. Our goal was to obtain as representative a picture of human physiology as possible; since we ultimately performed across-tissue analyses, we sought to avoid the inclusion of tissues whose presence could introduce gene expression similarities that would confound our tissue-specificity and coexpression analyses (see subsequent sections). As an example, we only included samples from subcutaneous and not from visceral adipose tissue.

We downloaded the raw RPKM data as provided in the GTEx portal. To determine whether a gene is expressed above a certain threshold or not, and for our tissue specificity and expression level analyses (Supplemental Methods), we used median(log_2_(RPKM + 1)). We assessed the impact of unwanted variation on the estimation of tissue specificity (Supplemental Fig. S18), as described in Supplemental Methods.

For our coexpression analysis, we downloaded the gene-level count table and transformed to the log_2_(RPM/10 + 1) scale (scaled to 10^7^ counts per sample instead of 10^6^). In this data set, five EM genes were not available, leaving us with 290 for analysis.

### Coexpression analysis

Using the GTEx data described above, we estimated tissue-specific networks and modules using the following approach. First, for each tissue, we only included genes for which the corresponding median expression (median(log_2_(RPKM + 1))) in that specific tissue was greater than zero. Then, prior to network construction, we preprocessed the expression data (on the log_2_(RPM/10 + 1) scale) to remove unwanted variation, which can confound the estimation of pairwise correlation coefficients between genes (Freytag et al. 2015; Parsana et al. 2017). Parsana et al. (2017) established that this can be addressed by removing leading principal components from the expression matrix. To avoid overfitting, we removed the same number of principal components from all tissues. In Supplemental Figure S19, we depict the impact of doing this on the distribution of pairwise correlations across (1) 2000 randomly selected genes, and (2) 80 genes encoding for the protein component of the ribosome (Supplemental Table S13), following ideas from Freytag et al. (2015). We expect random genes to be uncorrelated (negative controls), whereas we expect genes encoding for ribosomal proteins to be highly coexpressed (positive controls). Based on these assessments, we settled on removing four principal components.

We then proceeded to network construction. For each tissue, we estimated the soft thresholding power using the entire expression matrix, following standard WGCNA guidelines. To ensure that we can ultimately make comparisons across the 28 tissues, we next selected EM genes that have some minimal expression (median(log_2_(RPKM + 1)) > 0) in all of them. This gave us 270 EM genes. Subsequently, using only those 270 EM genes, we built unsigned tissue-specific networks and identified modules by performing hierarchical clustering using the function cutTreeDynamic(), with the dissimilarity measure based on the topological overlap matrix. We set the parameters minClusterSize and deepSplit equal to 15 and 2, respectively. Modules were merged when the correlation between the corresponding module eigengenes was 0.8 or greater. Any parameters that are not mentioned were left at their default values. We assessed the statistical significance of our results, the robustness to sample outlier, and the reproducibility with a different network inference method as described in Supplemental Methods.

### *Trans*-acting factor binding at EM gene promoters

We defined promoters as 10-kb sequences centered around the transcriptional start site. We used the ENCODE portal (https://www.encodeproject.org), to download TF ChIP-seq data for the K562 cell line (Supplemental Methods; Supplemental Table S14) and then selected genes expressed in K562 cells (Supplemental Methods). This yielded a total of 242 EM genes (72 highly coexpressed and 94 non-coexpressed), 14,355 other genes (excluding ribosomal protein genes), and 330 TFs. To test for enrichment of TF binding in the highly coexpressed versus the non-coexpressed EM gene group, we first discarded any TFs that were binding at only 10 promoters or less, as those were unlikely to be driving the observed coexpression. We then created a 2 × 2 table for each of the remaining 295 TFs, and performed Fisher's exact test. Finally, we examined the statistical significance of our results as described in Supplemental Methods.

### Stratified LD score regression

We used a list of brain enhancer regions defined by Vermunt et al. (2014) as genomic locations distinct from genic promoters, which are marked by H3K27ac in one or more of 136 brain samples from 87 anatomically distinct brain regions. We labeled each brain enhancer region within 1 Mb of the transcription start site (TSS) of an EM gene as an EM-regulatory region. This yielded 46 Mb of EM-regulatory regions and 15 Mb of highly coexpressed regulatory regions (with the former including the latter as a subset). We next used stratified LD score regression (SLDSR) (Bulik-Sullivan et al. 2015; Finucane et al. 2015) with the LDSC software (https://github.com/bulik/ldsc) to estimate coefficient *Z*-scores and enrichment statistics for these two sets of regions across 29 traits (Supplemental Methods; information regarding the corresponding GWAS studies is provided in Supplemental Table S11). The interpretation of the LDSC analysis is that a feature (a set of regions) is significantly enriched for explained heritability if the feature adds (significantly) to the explained heritability on top of the baseline model. Because the model includes (controls for) multiple types of features including coding and conserved regions, as well as LD structure, this is a stronger statement of enrichment than, for example, considering the enrichment of leading GWAS SNPs.

### Genome assembly version

All our analyses were performed using the GRCh37 (hg19) genome assembly version, primarily because the publicly available data sets we relied on (ExAC, as well as the GWAS data used in LDSC) utilized this genome version. Given that we focus on well-annotated regions of the genome, our results would not be significantly impacted by use of the newer GRCh38.

### Software availability

Analysis code for this work is available online (https://github.com/hansenlab/em_paper) and as Supplemental Code 1.

## Competing interest statement

H.T.B. is a paid consultant for Millennium Pharmaceuticals, Inc.

## Supplementary Material

Supplemental Material
