# Supplementary material for: Coexpression patterns define epigenetic regulators associated with neurological dysfunction
Source: Genome Res. 2019 Apr;29(4):532–42. doi: 10.1101/gr.239442.118 (PMC6442390; doi:10.1101/gr.239442.118)
Supplement: Supplemental Material [file supp_29_4_532__index.html]

Coexpression patterns define epigenetic regulators associated with neurological dysfunction — Supplemental Material 

# Coexpression patterns define epigenetic regulators associated with neurological dysfunction

## Supplemental Material

- Supplemental\_Code\_1.zip
- Supplemental\_Table\_S2.csv
- Supplemental\_Table\_S3.csv
- Supplemental\_Table\_S4.csv
- Supplemental\_Table\_S5.csv
- Supplemental\_Table\_S6.csv
- Supplemental\_Table\_S7.csv
- Supplemental\_Table\_S10.csv
- Supplemental\_Table\_S11.xlsx
- Supplemental\_Table\_S12.csv
- Supplemental\_Table\_S13.csv
- Supplemental\_Table\_S14.tsv
- Supplemental\_Materials.pdf
