# Supplementary material for: Coexpression patterns define epigenetic regulators associated with neurological dysfunction
Source: Genome Res. 2019 Apr;29(4):532–42. doi: 10.1101/gr.239442.118 (PMC6442390; doi:10.1101/gr.239442.118)
Supplement: Supplemental Material [file supp_gr.239442.118_Supplemental_Code_1.zip › ldsc/figures/Legend.pdf]

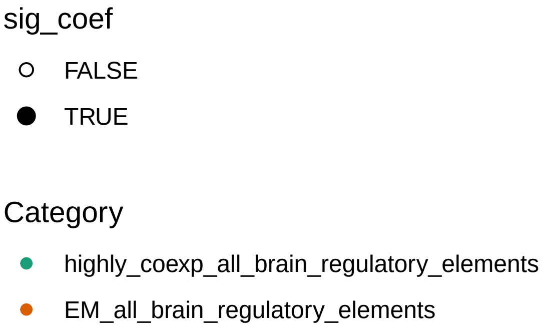
sig_coef
FALSE
TRUE
Category
highly_coexp_all_brain_regulatory_elements
EM_all_brain_regulatory_elements
