# Supplementary figures and images for: Coexpression patterns define epigenetic regulators associated with neurological dysfunction
Source: Genome Res. 2019 Apr;29(4):532–42. doi: 10.1101/gr.239442.118 (PMC6442390; doi:10.1101/gr.239442.118)

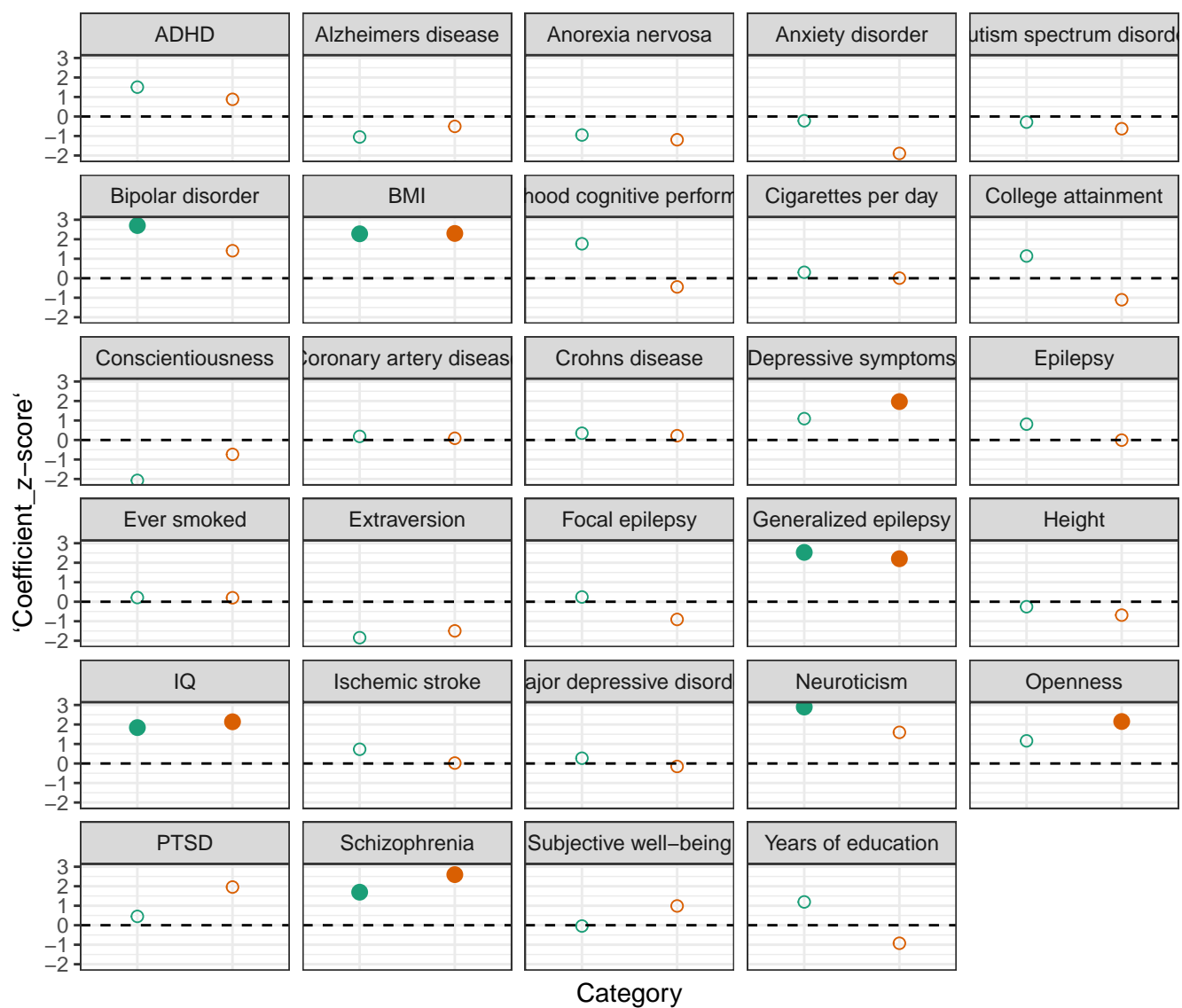

Supplement: Supplemental Material [file supp_gr.239442.118_Supplemental_Code_1.zip › ldsc/figures/Coefficient_Z-score.baseline_adjustments.pdf]

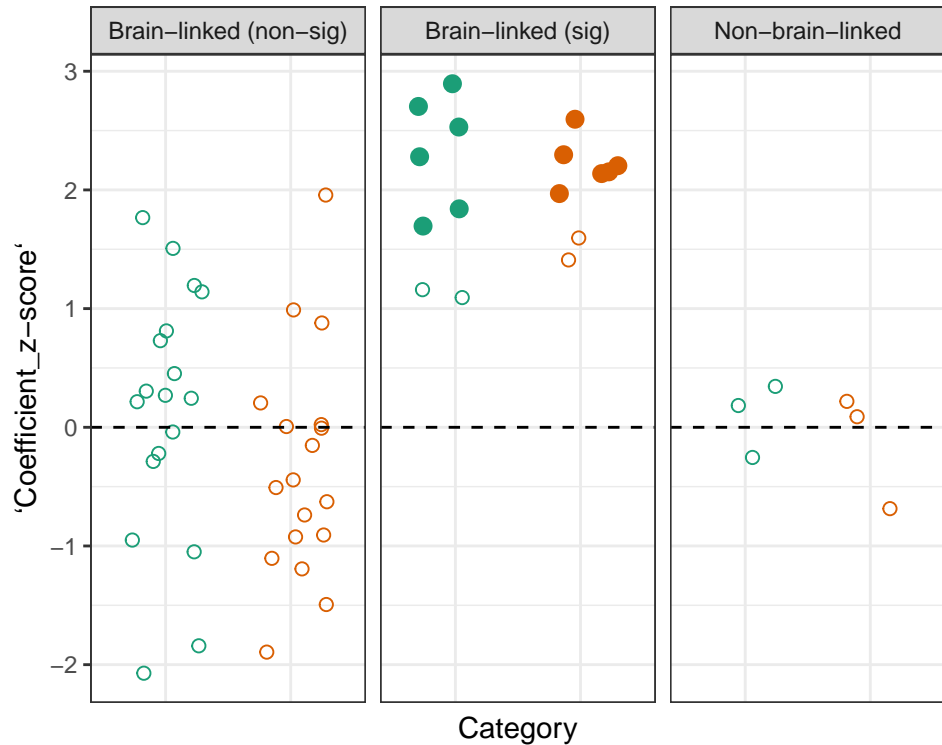

Supplement: Supplemental Material [file supp_gr.239442.118_Supplemental_Code_1.zip › ldsc/figures/Coefficient_Z-score.baseline_adjustments.stratified.pdf]

Enrichment

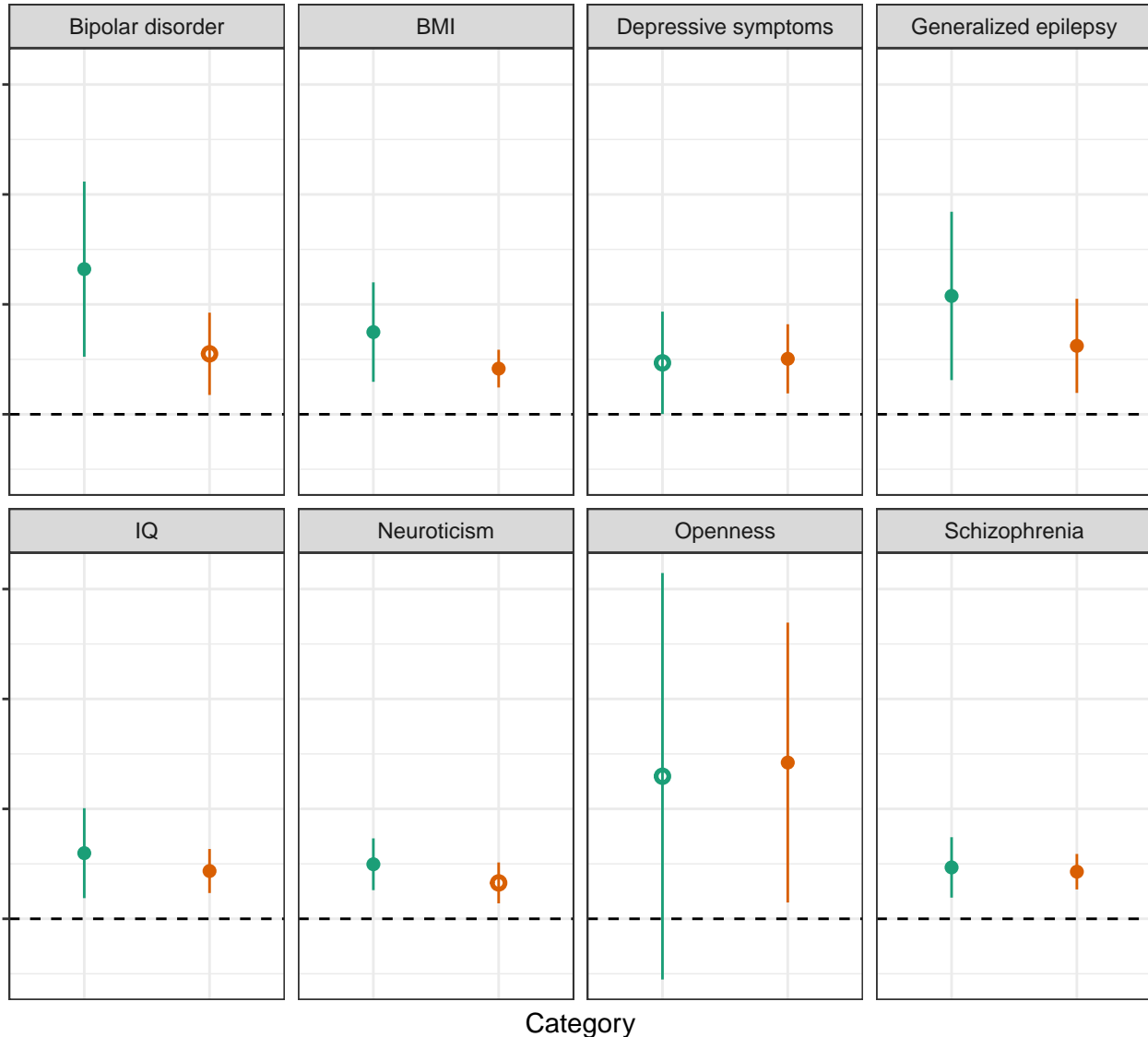

Supplement: Supplemental Material [file supp_gr.239442.118_Supplemental_Code_1.zip › ldsc/figures/Enrichment.baseline_adjustments.pdf]

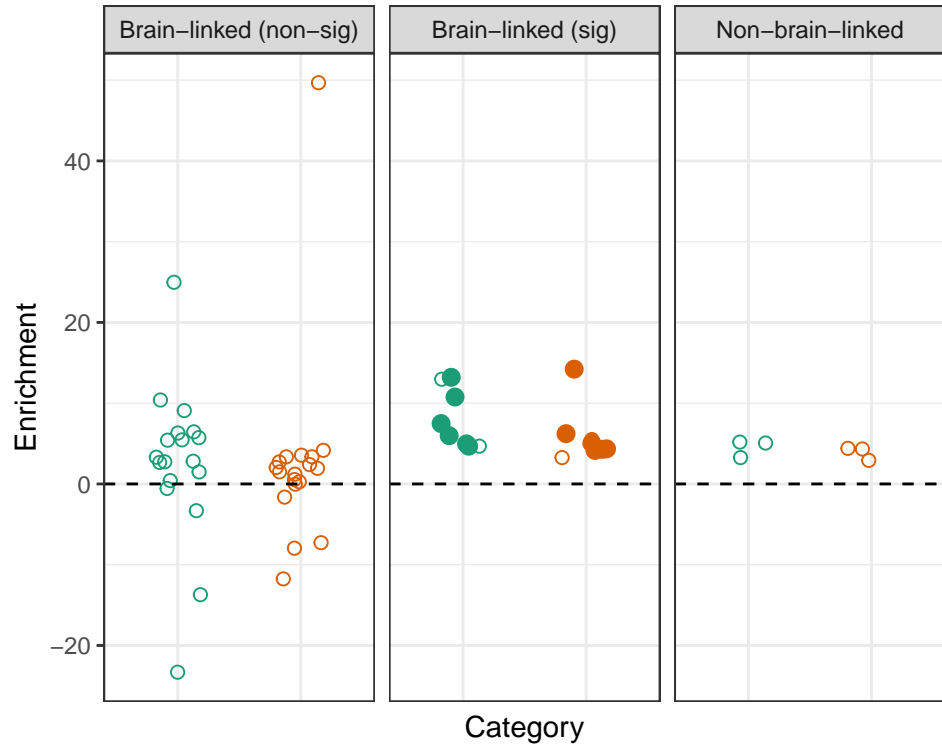

Supplement: Supplemental Material [file supp_gr.239442.118_Supplemental_Code_1.zip › ldsc/figures/Enrichment.baseline_adjustments.sig_stratified.pdf]
