# Supplementary material for: Coexpression patterns define epigenetic regulators associated with neurological dysfunction
Source: Genome Res. 2019 Apr;29(4):532–42. doi: 10.1101/gr.239442.118 (PMC6442390; doi:10.1101/gr.239442.118)
Supplement: Supplemental Material [file supp_gr.239442.118_Supplemental_Materials.pdf]

Supplemental Materials

for

Co-expression patterns define epigenetic regulators associated with neurological dysfunction

Leandros Boukas, James M. Havrilla, Peter F. Hickey, Aaron R. Quinlan,
Hans T. Bjornsson*, Kasper D. Hansen*

Contents

1. Supplemental Results
2. Supplemental Methods
3. Supplemental Tables S1-S14.
4. Supplemental Figures S1-S19.

*To whom correspondence should be addressed. Emails hbjorns1@jhmi.edu (HTB), khansen@jhsph.edu (KDH)

Supplemental Results

Overlap between the DNA methylation and the histone machinery

The overlap between the DNA methylation and histone components is multifaceted, and is due to: 1) 4 histone methyltransferases and 2 histone demethylases which also read methylated or unmethylated CpGs, 2) all 3 DNA methyltransferases which are histone methylation readers as well, and 3) 11 readers of histone acetylation and/or histone methylation which are also readers of methylated or unmethylated CpGs.

Variation intolerance of EM genes encoded on the sex chromosomes

In our main analysis of loss-of-function variation intolerance, we focused on genes encoded on the autosomes. When we exclusively considered the X chromosome, we observed a similar picture; 16 out of the 18 X-linked EM genes have a pLI greater than 0.9. Using data from a recent study on X inactivation (Tukiainen et al. 2017), we found that all 3 EM genes that consistently escape X inactivation in different tissues have a pLI of 1. In contrast, only 31% of other X-linked genes have a pLI greater than 0.9 (median pLI = 0.65, median pLI for other genes that escape X inactivation = 0.41). With respect to the 2 out of 4 EM genes on the Y chromosome that are included in ExAC, *UTY* has an intermediate pLI of 0.63, while *KDM5D* is haplosufficient (pLI = 0.02).

Variation intolerance of different types of epigenetic regulators

Although histone methyltransferases (HMTs) appear less constrained than histone acetyltransferases (HATs) (Fig. 2F,G), all but one of the HMTs also have a reader domain, and there is no difference in variation tolerance between dual-function HMTs and dual-function HATs (Wilcoxon rank sum test, $p = 0.61$). Two out of the three DNA methyltransferases (*DNMT1* and *DNMT3B*) are constrained (Fig. 2F,G), as is the case for DNA demethylases (with *TET2* being the only tolerant member, Fig. 2F,G), whereas histone demethylases and deacetylases are approximately evenly divided in the high and low pLI categories (Fig. 2F,G). Additionally, we found that all genes encoding for *CxxC* domain proteins, which recognize unmethylated CpG dinucleotides (Lee et al. 2001), show very high dosage sensitivity (median pLI = 0.97), with three of them being dual readers.

We also investigated the impact of the exact amino-acid substrate specificities of EM genes on their variation tolerance (Methods, Supplemental Table S8). Specifically, we examined writers/erasers of H3K4, H3K27, H3K36, and H3K9 methylation, and H3K27 acetylation writes and H3K9 acetylation erasers. More than 50% of genes in each category had a pLI$>$ 0.9 (Supplemental Fig. S2); this highlights that dosage sensitivity does not depend on the exact nucleosomal target position.

Tissue specificity and expression levels of EM genes

The intolerance to variation suggests that for most of the EM genes, the loss of even a single copy is incompatible with a healthy organismal state. An important question then, is the identification

of the tissues and cell types through which this detrimental effect is mediated. There are two primary reasons why one might speculate that some EM genes have tissue-specific expression. First, the composition of the machinery suggests the existence of functionally redundant components (for instance, there are 116 histone methylation readers with no enzymatic or other reading activity). This redundancy could be explained if different components with the same role were specific to different tissues. Second, there is some evidence suggesting that TF binding to their target sites requires, at least in some cases, an already permissive chromatin state (Guertin, Lis 2013). This could imply that there are certain EM components expressed in a cell-type specific fashion, that thereby help generate the epigenomic landscapes that facilitate TF binding (Guertin, Lis 2013; Quante, Bird 2016). Given that specific genomic locations need to be marked, it has been postulated that this is achieved by EM genes with DNA-binding domains that differ from those encountered in classical TFs, yet confer some degree of sequence specificity (Guertin, Lis 2013; Quante, Bird 2016). Four domains described as putative candidates are the ARID DNA-binding domain, the AT-hook DNA-binding motif, the CxxC domain, and the C2H2-like Zinc finger, which we found to be present in 21 EM genes.

To gain insight into the question of tissue-specificity, we examined the expression patterns of EM genes across a spectrum of adult tissues, using RNA seq data generated by the GTEx Consortium (The GTEx Consortium 2015). We selected 28 tissues on the basis of sample size and differences in physiological function, as this enabled us to obtain a comprehensive picture under diverse cellular conditions, and allowed us to avoid spurious specificity estimates arising from the high similarity between some tissues (e.g. subcutaneous and visceral adipose tissue). For each EM gene, we calculated an entropy based tissue-specificity score, as previously described (Cabili et al. 2011). This score reflects the degree to which a gene is highly specific for some tissue (score close to 1) or is expressed broadly across tissues (score close to 0). We discovered that the vast majority of EM genes are characterized by very low specificity, after comparing their scores to those of TF genes as well as other genes (Supplemental Fig. S6A). In fact, when we compared the specificity of EM genes to that of genes encoding for proteins involved in the tricarboxylic acid (TCA) cycle, a well known category of housekeeping genes, we found a very similar distribution (Supplemental Fig. S6B). The lack of tissue specificity characterizes EM genes with a DNA-binding domain that recognizes short motifs as well (median specificity score = 0.1), although we note that there also exist other genes that harbor those domains but do not fulfill the criteria for inclusion in our list of EM genes. This result however, raises the possibility that the increased dosage sensitivity of EM genes is due to their non-specific expression pattern. However, even after considering only highly non-specific genes (score less than 0.1), the enrichment of the 160 EM genes satisfying this criterion in the highly constrained category remains extremely pronounced (Supplemental Fig. S7). This is true both when comparing to all of the 5249 other non-specific genes (Fisher's exact test, $p < 2.2 \times 10^{-16}$, odds ratio = 5.6), as well as to the 232 non-specific TFs (Fisher's exact test, $p = 6.73 \times 10^{-9}$, odds ratio = 3.5), showing that this constraint is not merely a consequence of the presence of EM genes in a greater number of cell types, but rather reflects their function.

We subsequently reasoned that our analysis might be masking the presence of genes specific for only a small subset of tissues, and we performed a detailed analysis of the specificity of EM genes separately for each tissue (Supplemental Fig. S6C). We observed that testis stands out as the only tissue for which a small number of EM genes show specific expression (Supplemental Fig. S6C), indicating its dependence on not only the general machinery that operates in all other tissues,

but also on a distinct subset of components. This is in agreement with the existing view that testis is an outlier tissue with respect to its transcriptomic state (The GTEx Consortium 2015). The testis-specific EM genes include *PRDM9*, in accordance with its reported role in meiotic recombination (Hochwagen, Marais 2010), as well as 10 other genes (Supplemental Fig. S6C), some of which (*TDRD1*, *RNF17*, *BRDT*, *PRDM14*, *MORC1*) possibly play roles in male germ cell differentiation and the repression of transposable elements in the germline (Chuma et al. 2006; Pan et al. 2005; Shang et al. 2007; Yamaji et al. 2008; Pastor et al. 2014), while the role of the others (*CDY2A*, *HDGFL1*, *PRDM13*, *PRDM7*, *TDRD15*) remains mostly unspecified. Three of those genes are also members of the PRDM family of histone methyltransferases, while the rest are all readers of histone methylation, with the exception of *BRDT*, a histone acetylation reader.

Finally, in all of the tissues analyzed, we observe that EM genes are highly expressed compared to TF genes, and other genes (Supplemental Fig. S6D). We also confirm that TF genes are expressed at low levels (Supplemental Fig. S6D), as was previously observed using microarray data (Vaquerizas et al. 2009). Within EM genes however, the expression level does not robustly distinguish EM genes with high pLI (> 0.9) from the rest (Supplemental Fig. S8). As expected, there are exceptions, namely TF genes or other genes that are expressed at equally high or higher levels than EM genes, as well as a median of 44.5 EM genes across tissues expressed at low levels, with a median RPKM always less than 1. Within the latter, 9 components show consistently low expression across all tissues, with 6 being testis-specific. Collectively, the above results indicate that the majority of human EM genes are active across a heterogeneous set of adult tissues. This suggests that other factors primarily maintain cell identity in those tissues, and it can help explain the observation that in most cases of Mendelian disorders of the epigenetic machinery, more organ systems are affected compared to other genetic disorders (Bjornsson 2015).

Disease associations of epigenetic regulators

Consistent with previous observations (Bjornsson 2015), we found that neurological dysfunction is a very prevalent phenotype within those diseases. Specifically, a total of 50 out of the 101 disease-associated EM genes genes have been previously describe to lead to neurological dysfunction (Fig. 6B). Our analysis also yielded 64 EM genes associated with cancer (Fig. 6B). We highlight a substantial overlap between those two groups: 24 of cancer associated EM genes are also associated with neurological dysfunction (Fig. 6A), with dual function EM genes showing extremely high enrichment in this category (Fisher's exact test, $p = 1.84 \times 10^{-8}$, odds ratio = 13.3). We did not find any EM gene both associated with cancer and also causing a Mendelian disease, without neurological dysfunction being part of the disease phenotype.

Co-expressed epigenetic regulators are not spatially clustered

Given that highly expressed genes in the human genome tend to reside in chromosomal clusters (Caron et al. 2001), and taking into account that clustered genes are often co-expressed (Cohen et al. 2000), we investigated the relative chromosomal positions of co-expressed EM genes. We did not observe any notable clustering, with our 74 highly co-expressed, and 82 co-expressed EM genes being approximately uniformly distributed across chromosomes (chi-squared goodness

of fit test, $p = 1$ for both groups), and only 9 and 6 pairs, respectively, having a within-pair chromosomal distance less than 1 megabase (Supplemental Fig. S16). The single exception to this is *SETD1A* and *FBXL19*, which are separated by only 8 kb. To see if the co-expression of those two genes is driven by a bidirectional promoter we looked at whether they are encoded on opposite strands, and found this not to be the case.

The highly co-expressed epigenetic regulators are not enriched for protein-protein interactions

We tested whether the co-expression is associated with protein-protein interactions (PPIs) between EM gene products, using recent data on such interactions (Huttlin et al. 2017). As our definition of EM genes only includes these with catalytic or reading activity, and not genes encoding for accessory subunits of chromatin modifying complexes, the extent to which such interactions will occur is not *a priori* known. We did not observe increased frequency of interactions between the highly co-expressed versus the non co-expressed group, with both groups having very few PPIs (probability of a pair interacting is 0.0007 and 0.003 in the two groups respectively). This may suggest incomplete data on protein-protein interactions.

OMIM 2019

Between the time of analysis and the time of publication, 8 of our new disease candidate EM Genes were linked with a Mendelian phenotype (https://omim.org/). The MIM numbers of these phenotypes along with whether neurological dysfunction is part of the disease phenotype are included in Supplemental Table S6. Including these 8 genes when testing for enrichment in the highly co-expressed group produces very similar results (Fisher's exact test, $p = 0.002$, odds ratio = 3.5 for enrichment of EM genes associated with neurological dysfunction but not cancer).

Supplemental Methods

The creation of an epigenetic regulator list

We performed a manual literature curation to supplement our list of EM genes. We categorized all proteins belonging to the CHD family as chromatin remodelers (Marfella, Imbalzano 2007), and we included the two histone lysine demethylases that do not harbor the JmjC domain, KDM1A and KDM1B (Shi 2007). After manual curation, we excluded the proteins COIL, MSL3P1, ASH2L, PHF24, and VPRBP. We did not include the atypical histone lysine methyltransferase DOT1L, and we did not classify transcription factors whose recognition motifs include methylated CpG dinucleotides as DNA methylation readers. Proteins containing Ankyrin repeats were classified as histone methylation readers, provided they were first included as members of the epigenetic machinery based on the domains in Supplemental Table S1. We also note the case of the PHD finger domain: it was generally classified as a histone methylation reader domain, with the exception

of 5 proteins (DPF1,2, and 3, and KAT6A,B) that have a double PHD finger which, based on experimental evidence (Zeng et al. 2010; Huber et al. 2017) acts as a histone acetylation recognition mode.

Epigenetic regulators with disease associations

In addition to those associated with a Central Nervous System phenotype in OMIM, we also labeled the following EM genes as associated with neurological dysfunction: 1) genes that have been associated with Autism at a false discovery rate of 0.1 (De Rubeis et al. 2014) (those included the three genes later firmly associated with developmental disorders in Faundes et al. (2018)), 2) the top 15 % genes implicated in Schizophrenia (as ranked by their residual variation intolerance score in McCarthy et al. (2014)), 3) *SETD1A*(Singh et al. 2016), 4) *KMT2B* (Zech et al. 2016), 5) genes that lacked previous associations with developmental disorders but achieved genome-wide significance in the DDD study (Deciphering Developmental Disorders Study 2017).

CCR local constraint score

We manually mapped the genomic coordinates of the following protein domains not included in the Pbase package: 1) the ADD domain, 2) the 2OGFeDO, oxygenase domain, 3) the PWWP domain for *MUM1*, 4) the chromodomain for *MSL3*, 5) the PHD-fingers for *RAG2* and *UBR7*. This was done based on the amino acid coordinates provided by the InterPro website (`https://www.ebi.ac.uk/interpro/`).

Tissue specificity and expression level analysis

Using the GTEx data described above, we calculated tissue-specificity scores for Supplemental Fig. S6 as previously described (Cabili et al. 2011). Computations were done using the functions `makeprobs()` and `JSdistFromP()` from the cummeRbund package (Trapnell et al. 2012). Supplemental Figure S6B depicts the tissue-specificity scores of EM genes vs those of 30 genes encoding for TCA cycle related proteins (Supplemental Table S10). To confirm that our findings were not driven by unwanted variation, we repeated our analysis after correcting for RIN as well as surrogate variables (SVs) (Leek, Storey 2007; Leek, Storey 2008). In particular, using ($\log_2$(RPKM + 1)) values, we estimated the SVs using the function `sva()`, from the SVA R package (Leek, Johnson, et al. 2012), while protecting for the tissue effect and including RIN as a known confounder. This resulted in 182 significant SVs, which we then used along with RIN to obtain the corrected expression values using the function `removeBatchEffect()` in the limma R package (Ritchie et al. 2015). Subsequently, negative values in the expression matrix were replaced by zeros, and genes with uniformly low values (< 0.01 in all samples) were removed. As depicted in Supplemental Fig. S18), the results were essentially the same as those obtained with our original analysis. It should be mentioned however, that in situations where there is severe confounding of the factor of interest with some batch effect (for example, if samples from a particular tissue were all processed differently than others), correcting for surrogate variables cannot disentangle desired from undesired variation.

Co-expression analysis

For each tissue, we only included genes where the corresponding median expression ($\text{median}(\log_2(\text{RPKM} + 1))$) was greater than zero. To remove unwanted variation prior to co-expression network construction, for each tissue, we standardized the expression matrix (containing ($\log_2(\text{RPM} + 1)$) values) to have mean 0 and variance 1 across every gene, and removed the 4 leading principal components from this matrix by regressing on these PCs and then reconstructing a new matrix with the regression residuals, using the function `removePrincipalComponents()` in the WGCNA package (Zhang, Horvath 2005; Langfelder, Horvath 2008).

With respect to the estimation of the soft thresholding power, for each tissue we used the entire expression matrix (consisting of genes expressed above the threshold described above). We chose the first value for which the network was characterized by an approximately scale free topology, following standard WGCNA guidelines.

Our requirement for a minimal expression level in each of the 28 tissues ($\text{median}(\log_2(\text{RPKM} + 1)) > 0$) selected 270 EM genes out of 295; in addition to the 5 of the 295 EM genes are not present in GTEx (see above), it excludes the 11 EM genes which are testis-specific (testis-specificity score > 0.5), 7 genes that are either not expressed or expressed at very low levels in those tissues, and 2 genes (*DPF1* and *PHF21B*) that are expressed at a considerable level in more than 1 tissue but are very lowly expressed in some other tissues (*DPF1* was especially expressed in cerebral cortex and cerebellum, but this was not as pronounced after correcting for RIN and surrogate variables).

To derive the reference distribution of the number of highly co-expressed and co-expressed genes (Fig. 4D, Supplemental Fig. S11), we performed all of the steps described above, but, instead of EM genes, with 270 randomly selected genes; this was repeated 300 times. Because we observed that EM genes which belong to either the highly co-expressed or co-expressed group have higher expression across tissues than EM genes which are not co-expressed (Supplemental Fig. S10A), each time the random genes were sampled from the population of genes whose median expression level ($\text{median}(\log_2(\text{RPKM} + 1))$) was: (1) at least 0.5 in more than half of the tissues (11963 genes total), or (2) at least 3 in more than half of the tissues (5095 genes total). These two populations of genes have a similar expression level to the EM genes (group 1) or a considerably higher expression level compared to the EM genes (group 2) (see Supplemental Fig. S10B,C).

We examined the robustness of our results to the choice of arbitrary cutoffs, and determined that our choice of cutoffs do not impact the statistical significance of our finding (Supplemental Fig. S11). To ensure that our findings are not driven by the presence of outlier samples, we compared our result to those obtained when we randomly excluded samples from the network construction. In particular: 1) when there were more than 100 samples in a tissue, we randomly dropped half of them, 2) when there were between 50 and 100 samples in a tissue we randomly dropped 20 of them, and when there were less than 50 samples we randomly dropped 5 of them. After this subsampling had taken place, the subsequent steps were performed as described above. This procedure was repeated 300 times. Across the random subsets, we found a median of 64.5 out of the 74 originally identified highly co-expressed genes being classified as such again.

For the analysis where we construct tissue-specific networks by thresholding the correlation matrix, we selected genes and removed principal components as described above. We then estimated a tissue-specific threshold as the 99.8% percentile of the correlation matrix of 2,000 randomly cho-

sen genes. The topology of the resulting network is sensitive in both directions to the exact cutoff. We then thresholded the correlation matrix of the 270 EM genes and computed the maximally connected component of the resulting network. We then identified EM genes that shared membership in this component with more than 75 other EM genes in more than 10 tissues. We see 71 such genes, 60 of which are part of the originally detected highly co-expressed group, and 11 which are part of the co-expressed group, thus largely recapitulating the result obtained with WGCNA. We then compared the size and average node degree of the maximally connected component to those of the maximally connected components of networks constructed from groups of 270 randomly chosen genes; each of the reference distributions was derived by sampling 300 times from the population of genes with a similar expression level to EM genes (i.e. median expression greater than 0.5 in more than half the tissues, Supplemental Fig. S10). We found that EM gene networks had a significantly larger maximally connected component, whose average node degree was generally also larger (Supplemental Fig. S12).

Co-exression of transcription factors and protein kinases/phosphatases

To generate the reference distribution when assessing the co-expression of transcription factors, we randomly selected 915 genes whose median $\log_2(\text{RPKM}+1)$ expression was between 0.2 and 2.8 in more than half of the tissues, and repeated this 300 times. We then used the protein kinases provided in Manning et al. (2002), and excluded those labeled as pseudogenes, and the protein phosphatases with established catalytic activity, as provided in Chen et al. (2017). To generate the reference distribution when assessing the co-expression of protein kinases and phosphatases, we randomly selected 395 genes whose median $\log_2(\text{RPKM}+1)$ expression was at least 0.1 in more than half of the tissues, and repeated this 300 times.

Enrichment of disease categories in the highly co-expressed group

For Figure 6B we formed 2x2 tables of EM genes used in the co-expression analysis. For the categories "any disease", "neuro" and "ca", all 270 such genes were included. For the categories "neuro (no ca)", and "ca (no neuro)" all EM genes associated with both neurological dysfunction and cancer were excluded. In all cases compared EM genes in the highly co-expressed group to EM genes in either the co-expressed or the not co-expressed group (combined). For the "high pLI neuro vs. other high pLI" category we only included genes with a pLI greater than 0.9 (without excluding those on the sex chromosomes). For Figure 6C we compared EM genes in the highly co-expressed group (defined using different cutoffs) to EM genes in the not co-expressed group, keeping the latter reference group constant in all comparisons.

Trans-acting factor binding at EM gene promoters

We defined promoters as 10 kb sequences centered around the transcriptional start site. We used the ENCODE portal (http://encodeproject.org), to download TF ChIP-seq data for the K562 cell line (we note here that those data provide information for transcription factors, as well as other regulators not strictly belonging to the TF group; for simplicity, in this section we will refer to all

those factors as TFs). To take antibody quality into account, we followed the ENCODE guidelines and selected experiments that showed reproducibility across replicates and had narrow peak calls (that is, whose output type was labeled as ”optimal IDR thresholded peaks”). Then, we only kept experiments performed in the absence of any treatment on the cells. We also randomly discarded any duplicate experiments (that is, experiments performed on the same TF target, regardless of the exact antibody used). Subsequently, we used the recount R package (Collado-Torres, Nellore, Kammers, et al. 2017; Collado-Torres, Nellore, Jaffe 2017) to select genes expressed in K562 cells (Slavoff et al. (2013); study identifier in the sequence read archive: SRP010061); as in our co-expression analysis, we required that a gene had median $RPKM > 0$ across the 8 total samples. This yielded a total of 242 EM genes (72 highly co-expressed and 94 non co-expressed), 14355 other genes (excluding ribosomal protein genes), and 330 regulatory factors.

To test for enrichment of TF binding in the highly co-expressed versus the non co-expressed EM gene group, we first discarded any TFs that were binding at only 10 promoters or less, as those were unlikely to be driving the observed co-expression. We then formed a $2x2$ table for each of the remaining 295 TFs, and performed Fisher's exact test. To derive a null distribution we used the following two approaches. First, we initially split the set of 14355 other genes to a set of 9495 genes with a median $RPKM > 1.2$ and a set of 10821 genes with a median $RPKM > 0.4$, to match the expression levels of the highly co-expressed and non co-expressed EM genes respectively (Supplemental Fig. S17A). Then, we randomly sampled from these two sets to create two groups, consisting of 72 and 94 members respectively. We discarded any TFs binding at 50 promoters or less and then, as before, we tested each of the remaining 320 TFs for enrichment. Supplemental Fig. S17B depicts the null distribution of the number of TFs with an Odds Ratio > 2 (indicating at least a 2-fold enrichment in the 72-member group) and a p-value < 0.05, versus the observed number of TFs showing this enrichment in the highly co-expressed EM group. For the second approach, we randomly sampled EM genes and after sampling we arbitrarily created two groups, one with 72 members and another with 94. We then repeated the same procedure as before, and tested each TF for enrichment in the promoters of one group versus the other. Supplemental Fig. S17C shows the resulting null distribution, as well as the actual observed value.

Stratified LD score regression

For a given trait, Stratified LD Score Regression estimates the proportion of heritability that is explained by SNPs that reside within a given set of genomic locations. It employs a linear model that incorporates GWAS summary statistics as well as linkage disequilibrium values (as derived from a reference panel matched for ancestry). Each of our 2 sets of features (the highly co-expressed and the all-EM regulatory regions; see Methods) was separately examined for heritability enrichment, after adjusting for the full baseline set of features described in Bulik-Sullivan et al. (2015). This includes standard features such as coding regions and conserved regions. The resulting p-values associated with the z-scores were corrected for multiple testing using Holm's method (Holm 1979), within each of the 29 traits and across the 2 sets of features we considered.

References

Bjornsson HT. 2015. The Mendelian disorders of the epigenetic machinery. *Genome Res* **25**: 1473–1481.

Bulik-Sullivan BK, Loh PR, Finucane HK, Ripke S, Yang J, Schizophrenia Working Group of the Psychiatric Genomics Consortium, Patterson N, Daly MJ, Price AL, Neale BM. 2015. LD Score regression distinguishes confounding from polygenicity in genome-wide association studies. *Nat Genet* **47**: 291–295.

Cabili MN, Trapnell C, Goff L, Koziol M, Tazon-Vega B, Regev A, Rinn JL. 2011. Integrative annotation of human large intergenic noncoding RNAs reveals global properties and specific subclasses. *Genes Dev* **25**: 1915–1927.

Caron H, Schaik B van, Mee M van der, Baas F, Riggins G, Sluis P van, Hermus MC, Asperen R van, Boon K, Voûte PA, et al. 2001. The human transcriptome map: clustering of highly expressed genes in chromosomal domains. *Science* **291**: 1289–1292.

Chen MJ, Dixon JE, Manning G. 2017. Genomics and evolution of protein phosphatases. *Sci Signal* **10**: eaag1796.

Chuma S, Hosokawa M, Kitamura K, Kasai S, Fujioka M, Hiyoshi M, Takamune K, Noce T, Nakatsuji N. 2006. Tdrd1/Mtr-1, a tudor-related gene, is essential for male germ-cell differentiation and nuage/germinal granule formation in mice. *Proc Natl Acad Sci* **103**: 15894–15899.

Cohen BA, Mitra RD, Hughes JD, Church GM. 2000. A computational analysis of whole-genome expression data reveals chromosomal domains of gene expression. *Nat Genet* **26**: 183–186.

Collado-Torres L, Nellore A, Jaffe AE. 2017. recount workflow: Accessing over 70,000 human RNA-seq samples with Bioconductor. *F1000Res* **6**: 1558.

Collado-Torres L, Nellore A, Kammers K, Ellis SE, Taub MA, Hansen KD, Jaffe AE, Langmead B, Leek JT. 2017. Reproducible RNA-seq analysis using recount2. *Nat Biotechnol* **35**: 319–321.

De Rubeis S, He X, Goldberg AP, Poultney CS, Samocha K, Cicek AE, Kou Y, Liu L, Fromer M, Walker S, et al. 2014. Synaptic, transcriptional and chromatin genes disrupted in autism. *Nature* **515**: 209–215.

Deciphering Developmental Disorders Study. 2017. Prevalence and architecture of de novo mutations in developmental disorders. *Nature* **542**: 433–438.

Faundes V, Newman WG, Bernardini L, Canham N, Clayton-Smith J, Dallapiccola B, Davies SJ, Demos MK, Goldman A, Gill H, et al. 2018. Histone Lysine Methylases and Demethylases in the Landscape of Human Developmental Disorders. *Am J Hum Genet* **102**: 175–187.

Guertin MJ, Lis JT. 2013. Mechanisms by which transcription factors gain access to target sequence elements in chromatin. *Curr Opin Genet Dev* **23**: 116–123.

Hochwagen A, Marais GAB. 2010. Meiosis: a PRDM9 guide to the hotspots of recombination. *Curr Biol* **20**: R271–R274.

Holm S. 1979. A simple sequentially rejective multiple test procedure. *Scandinavian journal of statistics*: 65–70.

Huber FM, Greenblatt SM, Davenport AM, Martinez C, Xu Y, Vu LP, Nimer SD, Hoelz A. 2017. Histone-binding of DPF2 mediates its repressive role in myeloid differentiation. *Proc Natl Acad Sci* **114**: 6016–6021.

Huttlin EL, Bruckner RJ, Paulo JA, Cannon JR, Ting L, Baltier K, Colby G, Gebreab F, Gygi MP, Parzen H, et al. 2017. Architecture of the human interactome defines protein communities and disease networks. *Nature* **545**: 505–509.

Langfelder P, Horvath S. 2008. WGCNA: an R package for weighted correlation network analysis. *BMC Bioinformatics* **9**: 559.

Lee JH, Voo KS, Skalnik DG. 2001. Identification and characterization of the DNA binding domain of CpG-binding protein. *J Biol Chem* **276**: 44669–44676.

Leek JT, Johnson WE, Parker HS, Jaffe AE, Storey JD. 2012. The sva package for removing batch effects and other unwanted variation in high-throughput experiments. *Bioinformatics* **28**: 882–883.

Leek JT, Storey JD. 2007. Capturing heterogeneity in gene expression studies by surrogate variable analysis. *PLOS Genet* **3**: 1724–1735.

Leek JT, Storey JD. 2008. A general framework for multiple testing dependence. *Proc Natl Acad Sci* **105**: 18718–18723.

Manning G, Whyte DB, Martinez R, Hunter T, Sudarsanam S. 2002. The protein kinase complement of the human genome. *Science* **298**: 1912–1934.

Marfella CGA, Imbalzano AN. 2007. The Chd family of chromatin remodelers. *Mutat Res* **618**: 30–40.

McCarthy SE, Gillis J, Kramer M, Lihm J, Yoon S, Berstein Y, Mistry M, Pavlidis P, Solomon R, Ghiban E, et al. 2014. De novo mutations in schizophrenia implicate chromatin remodeling and support a genetic overlap with autism and intellectual disability. *Mol Psychiatry* **19**: 652–658.

Pan J, Goodheart M, Chuma S, Nakatsuji N, Page DC, Wang PJ. 2005. RNF17, a component of the mammalian germ cell nuage, is essential for spermiogenesis. *Development* **132**: 4029–4039.

Pastor WA, Stroud H, Nee K, Liu W, Pezic D, Manakov S, Lee SA, Moissiard G, Zamudio N, Bourc'his D, et al. 2014. MORC1 represses transposable elements in the mouse male germline. *Nat Commun* **5**: 5795.

Quante T, Bird A. 2016. Do short, frequent DNA sequence motifs mould the epigenome? *Nat Rev Mol Cell Biol* **17**: 257–262.

Ritchie ME, Phipson B, Wu D, Hu Y, Law CW, Shi W, Smyth GK. 2015. limma powers differential expression analyses for RNA-sequencing and microarray studies. *Nucleic Acids Res* **43**: e47.

Shang E, Nickerson HD, Wen D, Wang X, Wolgemuth DJ. 2007. The first bromodomain of Brdt, a testis-specific member of the BET sub-family of double-bromodomain-containing proteins, is essential for male germ cell differentiation. *Development* **134**: 3507–3515.

Shi Y. 2007. Histone lysine demethylases: emerging roles in development, physiology and disease. *Nat Rev Genet* **8**: 829–833.

Singh T, Kurki MI, Curtis D, Purcell SM, Crooks L, McRae J, Suvisaari J, Chheda H, Blackwood D, Breen G, et al. 2016. Rare loss-of-function variants in SETD1A are associated with schizophrenia and developmental disorders. *Nat Neurosci* **19**: 571–577.

Slavoff SA, Mitchell AJ, Schwaid AG, Cabili MN, Ma J, Levin JZ, Karger AD, Budnik BA, Rinn JL, Saghatelian A. 2013. Peptidomic discovery of short open reading frame-encoded peptides in human cells. *Nat Chem Biol* **9**: 59–64.

The GTEx Consortium. 2015. Human genomics. The Genotype-Tissue Expression (GTEx) pilot analysis: multitissue gene regulation in humans. *Science* **348**: 648–660.

Trapnell C, Roberts A, Goff L, Pertea G, Kim D, Kelley DR, Pimentel H, Salzberg SL, Rinn JL, Pachter L. 2012. Differential gene and transcript expression analysis of RNA-seq experiments with TopHat and Cufflinks. *Nat Protoc* **7**: 562–578.

Tukiainen T, Villani AC, Yen A, Rivas MA, Marshall JL, Satija R, Aguirre M, Gauthier L, Fleharty M, Kirby A, et al. 2017. Landscape of X chromosome inactivation across human tissues. *Nature* **550**: 244–248.

Vaquerizas JM, Kummerfeld SK, Teichmann SA, Luscombe NM. 2009. A census of human transcription factors: function, expression and evolution. *Nat Rev Genet* **10**: 252–263.

Yamaji M, Seki Y, Kurimoto K, Yabuta Y, Yuasa M, Shigeta M, Yamanaka K, Ohinata Y, Saitou M. 2008. Critical function of Prdm14 for the establishment of the germ cell lineage in mice. *Nat Genet* **40**: 1016–1022.

Zech M, Boesch S, Maier EM, Borggraefe I, Vill K, Laccone F, Pilshofer V, Ceballos-Baumann A, Alhaddad B, Berutti R, et al. 2016. Haploinsufficiency of KMT2B, Encoding the Lysine-Specific Histone Methyltransferase 2B, Results in Early-Onset Generalized Dystonia. en. *Am J Hum Genet* **99**: 1377–1387.

Zeng L, Zhang Q, Li S, Plotnikov AN, Walsh MJ, Zhou MM. 2010. Mechanism and regulation of acetylated histone binding by the tandem PHD finger of DPF3b. *Nature* **466**: 258–262.

Zhang B, Horvath S. 2005. A general framework for weighted gene co-expression network analysis. *Stat Appl Genet Mol Biol* **4**: Article17.

Supplemental Tables

List of Supplemental Tables

S1 **Supplemental Table S1**: The EM-specific protein domains used to define the components of the epigenetic machinery.

S2 **Supplemental Table S2 [Available online]**: The components of the epigenetic machinery. An online version is available at `http://www.epigeneticmachinery.org`. A detailed description of columns in this file is given below.

S3 **Supplemental Table S3 [Available online]**: Local CCR constraint for all domains (rows), for all genes included in the local constraint analysis.

S4 **Supplemental Table S4 [Available online]**: The 38 EM genes with DNA binding domains. These include: 1) the 20 genes which are also classified as TFs under the "TF_activity" column in Supplemental Table S2, 2) EM genes containing a CxxC-type Zinc finger, or a High-mobility group box domain, or an AT-hook DNA-binding motif.

S5 **Supplemental Table S5 [Available online]**: A list of the accessory and EM subunits of the 19 complexes involving EM genes.

S6 **Supplemental Table S6 [Available online]**: Novel EM disease candidate genes, along with their co-expression status. We also provide the phenotype MIM number and the association with neurological dysfunction (if present) for 8 of these that had been associated with Mendelian phenotypes in OMIM at the time of publication.

S7 **Supplemental Table S7 [Available online]**: Novel disease candidate genes encoding for accessory subunits of EM complexes.

S8 **Supplemental Table S8**: EM histone modifiers for which the amino-acid substrate specificities are known.

S9 **Supplemental Table S9**: GTEx tissues used for the analyses.

S10 **Supplemental Table S10 [Available online]**: The 30 genes encoding for TCA cycle related proteins.

S11 **Supplemental Table S11 [Available online]**: The 29 common traits for which stratified LD-score regression was performed. The table includes the sample size for each GWAS, as well as links to the summary statistics.

S12 **Supplemental Table S12 [Available online]**: Partitioning of the genomic segments overlapping protein domains of EM genes into regions with different local constraint according to the CCR model.

S13 **Supplemental Table S13 [Available online]**: 80 genes encoding for protein components of the ribosome.

S14 **Supplemental Table S14 [Available online]**: Metadata relevant to the TF ChIP-seq experiments used in the trans-acting factor binding analysis.

Supplemental Table S1. The protein domains used to define the epigenetic machinery.

Domain name	Interpro ID
SET domain	IPR000182
GNAT domain	IPR000313
Histone acetyltransferase domain, MYST-type	IPR001025
Histone acetyltransferase Rtt109/CBP	IPR001214
JmjC domain	IPR001487
Histone deacetylase domain	IPR001680
Sirtuin family & catalytic core domain	IPR001739
Chromo domain	IPR001965
Zinc finger & PHD-type	IPR002110
Tudor domain	IPR002717
PWWP domain	IPR002857
Protein ASX-like & PHD domain	IPR002999
Bromo adjacent homology (BAH) domain	IPR003347
ADD domain	IPR004092
Mbt repeat	IPR011124
Zinc finger, CW-type	IPR013178
Bromodomain	IPR025766
Zinc finger, CXXC-type	IPR026590
Methyl-CpG DNA binding	IPR026905

Supplemental Table S8. Histone modifiers for which the amino-acid substrate specificity is known.

Specificity	Gene name
H3K4 methylation writer	KMT2A, KMT2B, KMT2C, KMT2D, SETD1A, SETD1B, SETD7, SMYD1, SMYD2, ASH1L, PRDM9
H3K27 methylation writer	EZH1, EZH2
H3K36 methylation writer	NSD1, WHSC1, WHSC1L1, SETD2, SMYD2, ASH1L, SETD3, SETMAR
H3K9 methylation writer	PRDM2, EHMT1, EHMT2, SETDB1, SUV39H1
H3K4 methylation eraser	KDM1A, KDM1B, KDM5A, KDM5B, KDM5C, KDM5D, NO66
H3K27 methylation eraser	KDM6A, UTY, KDM6B, KDM7A, PHF8
H3K36 methylation eraser	KDM2A, KDM2B, KDM4A, KDM4B, KDM4C, KDM4D
H3K9 methylation eraser	KDM3A, KDM3B, JMJD1C, KDM4A, KDM4B, KDM4C, KDM4D, PHF8, PHF2
H3K27 acetylation writer	EP300, CREBBP
H3K9 acetylation eraser	SIRT1, SIRT2

Supplemental Table S9. GTEx tissues used in the tissue specificity and co-expression analyses.

**Tissue name**	
Adipose – Subcutaneous,	Adrenal Gland,
Artery – Tibial,	Brain – Cerebellum,
Brain – Cortex,	Breast – Mammary Tissue,
Colon – Transverse,	Esophagus – Mucosa,
Heart – Left Ventricle,	Kidney - Cortex,
Liver,	Lung,
Minor Salivary Gland,	Muscle – Skeletal,
Nerve – Tibial,	Ovary,
Pancreas,	Pituitary,
Prostate,	Skin – Not Sun Exposed (Suprapubic),
Small Intestine – Terminal Ileum,	Spleen,
Stomach,	Testis,
Thyroid,	Uterus,
Vagina,	Whole Blood

Supplemental Figures

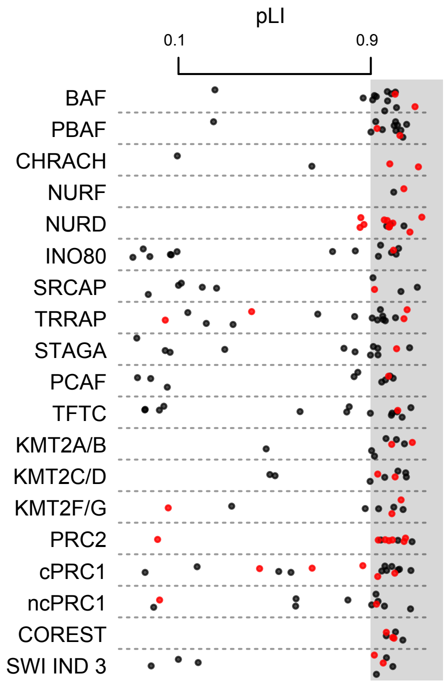

Supplemental Figure S1. The pLI scores of members of EM protein complexes. The pLI scores of EM (red points) and accessory (black points) subunits, depicted separately within each of 19 EM protein complexes (Methods). The grey area indicates genes with a pLI > 0.9.

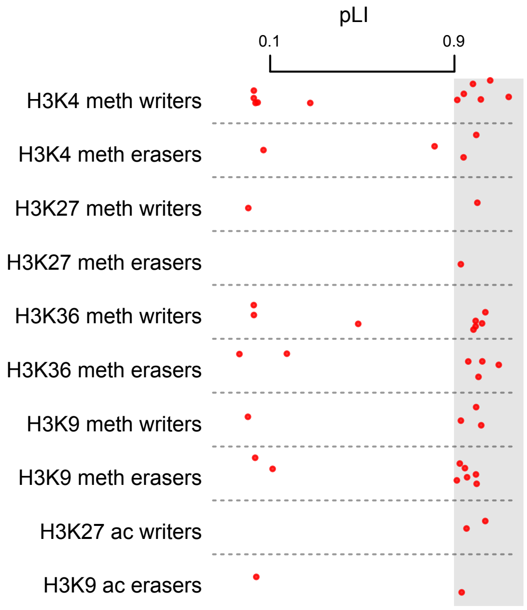

Supplemental Figure S2. pLI for EM genes with same substrate specificity. The pLI scores of EM genes grouped according to amino-acid substrate specificity, for the EM genes where this specificity is well defined. Only genes on autosomes are included.

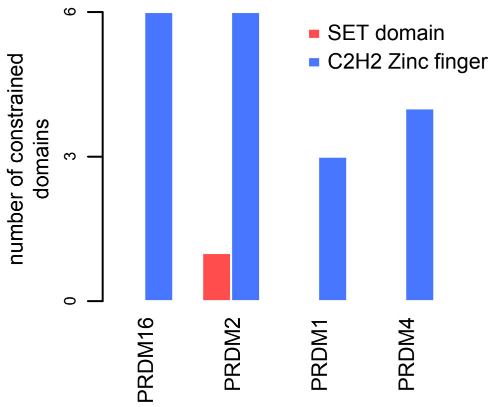

Supplemental Figure S3. The C2H2 zinc fingers are the main drivers of the mutational constraint of the PRDM family. Depicted are the four members of the PRDM family with high pLI, that contain both a SET domain as well as C2H2 zinc fingers. (In total, there are 15 PRDM members that are EM genes. 5 have a high pLI, and for one of them (*PRDM10*), the SET domain is not annotated by the Pbase package (see also Methods))

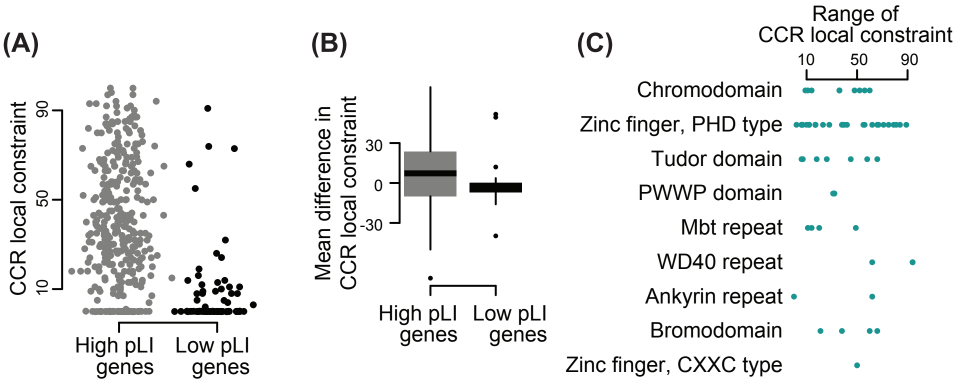

Supplemental Figure S4. The protein domains known to mediate epigenetic functions drive the observed constraint of EM genes. (A) The CCR local constraint of all EM-specific protein domains for high pLI (> 0.9) EM genes (grey points) and low pLI (< 0.1) EM genes (black points). The two groups are significantly different ($p < 2.2 \times 10^{-16}$, one-sided Wilcoxon rank-sum test). **(B)** The distribution of within-gene differences in the average CCR local constraint of EM-specific domains minus the average CCR local constraint of non EM-specific domains for high pLI EM genes (grey box; paired t-test, $p = 0.02$) and low pLI EM genes (black box; paired t-test, $p = 0.95$). **(C)** EM reader domains that appear in more than 1 copy within the same gene show within-gene variability in CCR local constraint (each point corresponds to the range of CCR local constraint scores for the different copies of a domain within the same gene; only data for high pLI EM genes are shown).

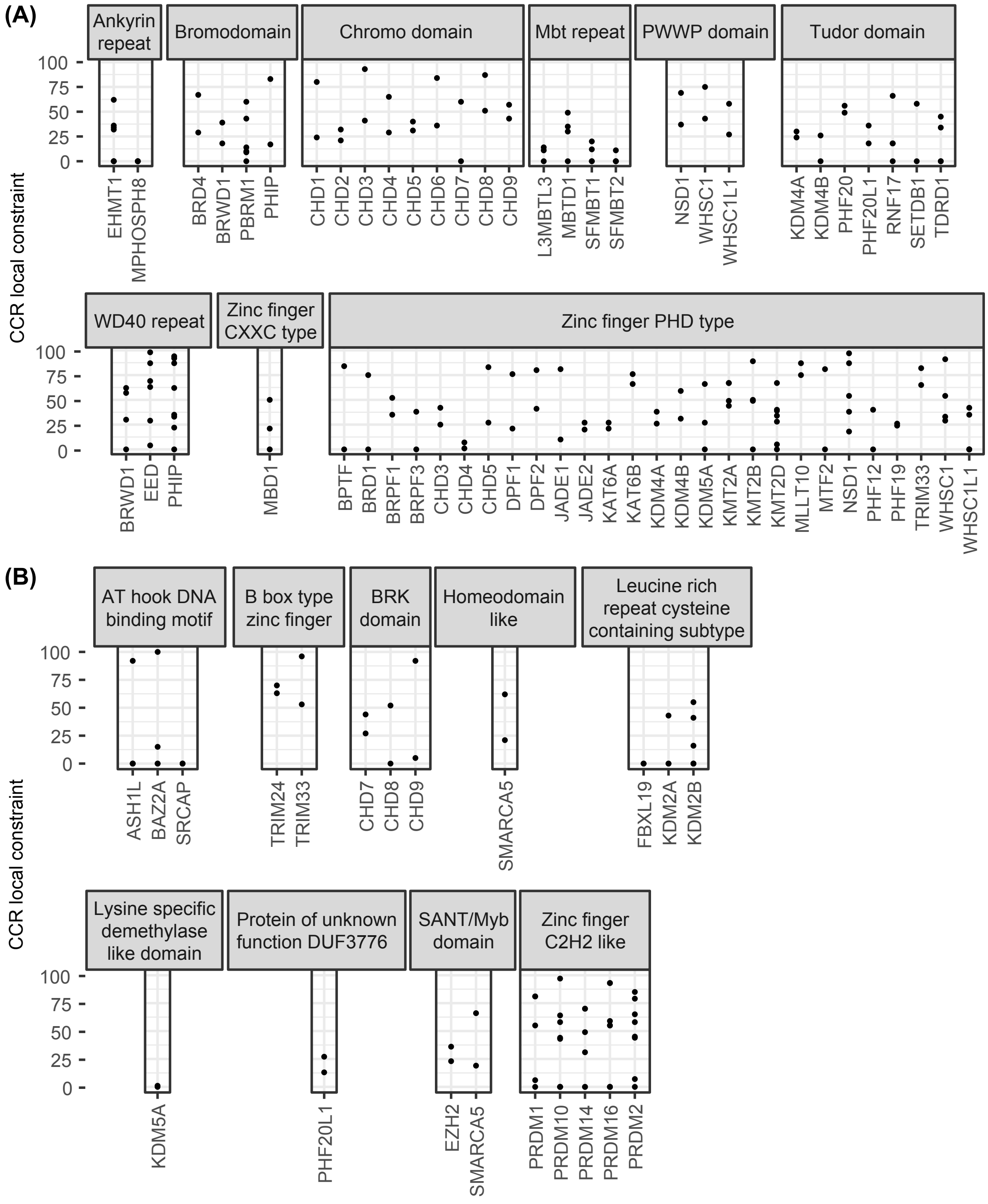

Supplemental Figure S5. Identical copies of protein domains show within-gene variability in constraint. Each plot corresponds to a domain, and the points therein are the CCR local constraint scores for the different copies of the same domain within each gene. Only genes with more than one copy of the particular domain are shown. **(A)** EM-specific domains. **(B)** non EM-specific domains in EM genes.

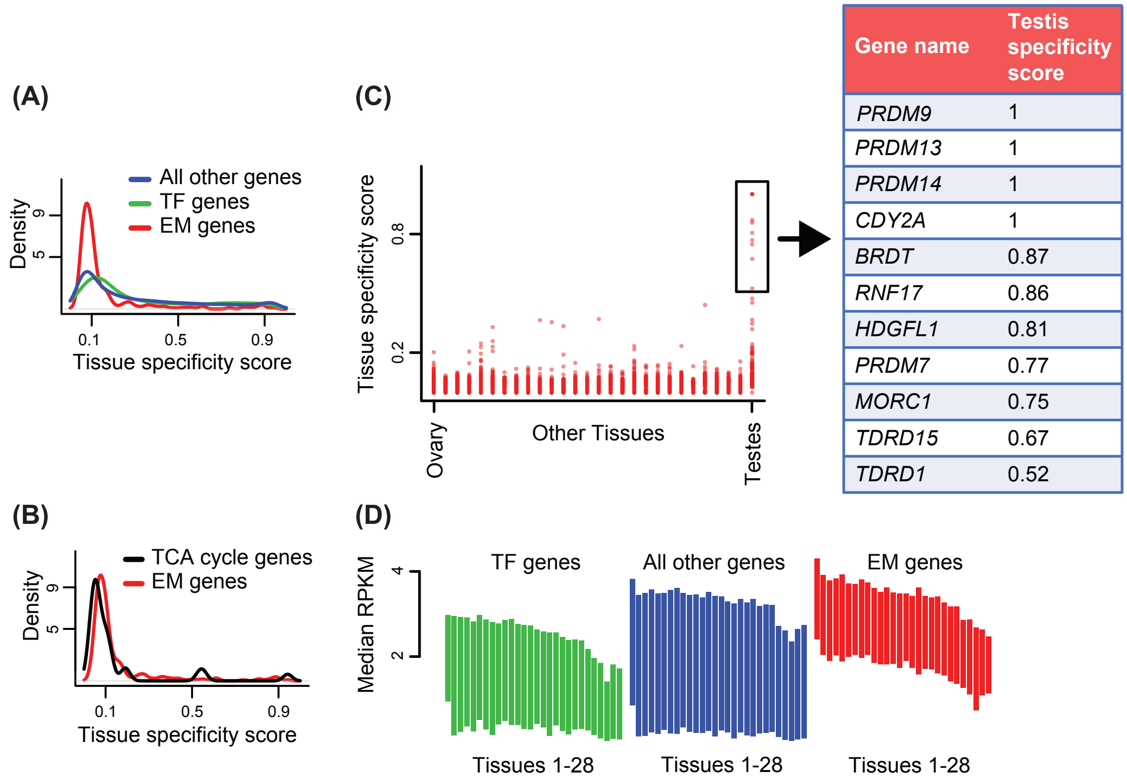

Gene name	Testis specificity score
PRDM9	1
PRDM13	1
PRDM14	1
CDY2A	1
BRDT	0.87
RNF17	0.86
HDGFL1	0.81
PRDM7	0.77
MORC1	0.75
TDRD15	0.67
TDRD1	0.52

Supplemental Figure S6. The components of the epigenetic machinery are expressed in a highly non tissue-specific manner and at high levels across tissues. (A) The distribution of the tissue-specificity score of EM genes (red curve) reveals their lack of tissue-specific expression, compared to TF genes (green curve), and all other genes (blue curve). **(B)** Comparison of the tissue-specificity of EM genes (red curve) with that of genes encoding for tricarboxylic acid (TCA) cycle related proteins (black curve) shows that EM genes exhibit comparable tissue-specificity to this class of well known housekeeping genes. **(C)** Testis is the sole tissue for which some EM genes have high specificity. **(D)** A comparison of expression levels of EM genes (red boxes) to those of TF genes (green boxes) and all other genes (blue boxes) shows their high relative expression. Each box shows the inter-quartile range of expression values, and tissues are ordered according to median expression for EM genes.

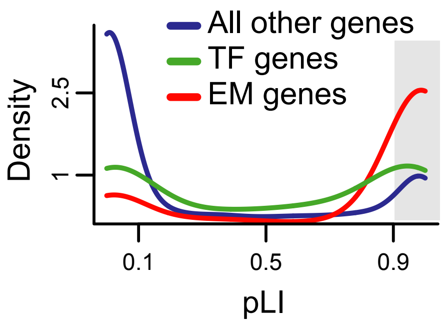

Supplemental Figure S7. The pLI distributions of genes with low tissue-specificity. Density plots of pLI scores for genes with low tissue-specificity score (< 0.1) highlight that the enrichment of EM genes (red curve) in the highly intolerant category (pLI > 0.9, gray shaded area) compared to TF genes (green) and other genes (blue) remains very pronounced even after considering only broadly expressed genes.

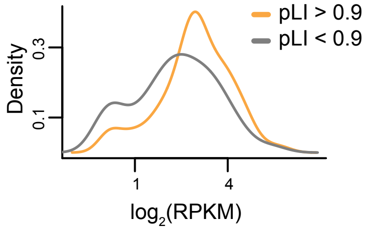

Supplemental Figure S8. The expression levels of high pLI EM genes are only weakly higher than of the rest. Density plots of $\log 2(\text{RPKM})$ values for EM genes with pLI > 0.9 vs. those of EM genes with pLI < 0.9.

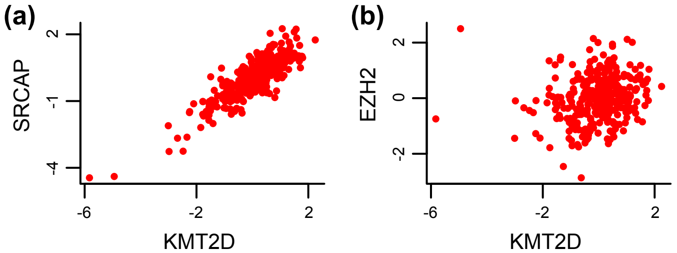

Supplemental Figure S9. EM genes show inter-individual variability in their expression levels. Data from subcutaneous adipose tissue from GTEx on 348 individuals. **(A)** Scatterplot of the expression levels of two EM genes (*KMT2D* and *SRCAP*) whose expression across individuals is highly correlated. **(B)** Scatterplot of the expression levels of two EM genes (*KMT2D* and *EZH2*) whose expression across individuals is uncorrelated.

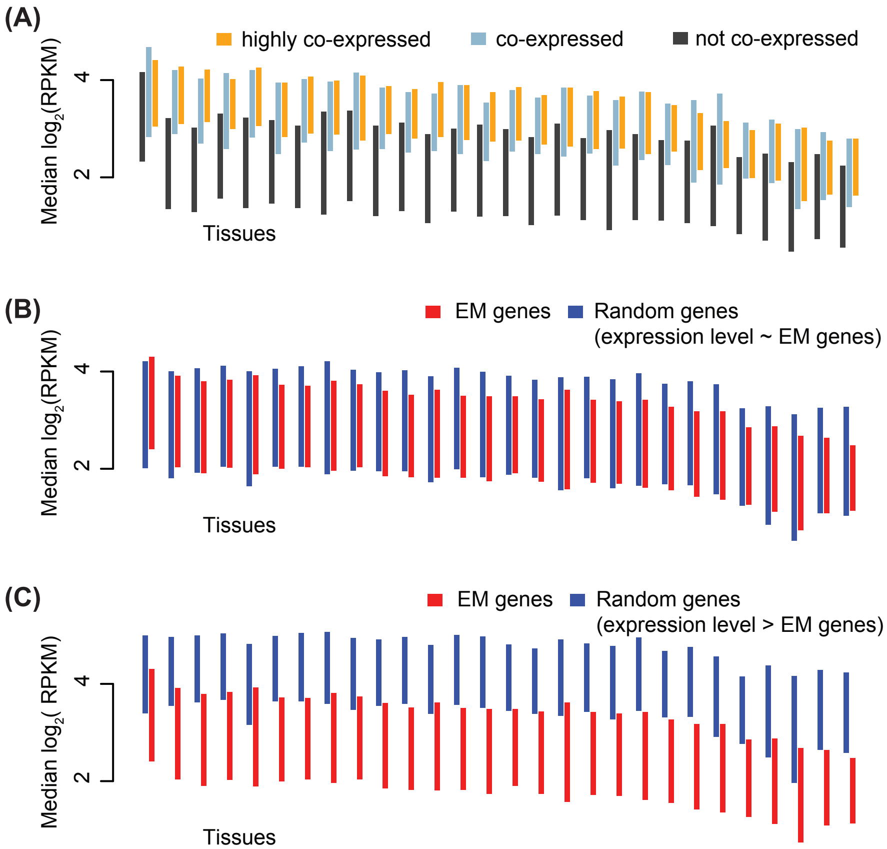

Supplemental Figure S10. Expression levels of EM genes. Expression (log(RPKM + 1))) for various groups of genes. **(A)** We categorize EM genes into 3 groups based on co-expressed module patterns across tissues (Fig. 4). EM genes which are highly co-expressed or co-expressed have a higher expression level than EM genes which are not co-expressed. The former two categories show similar expression levels. **(B)** The expression level of EM genes compared to 11,963 genes where the median expression in each tissue is greater than 0.5 in more than half the tissues. The two groups of genes have similar expression level. We say these genes are similarly expressed to the EM genes. **(C)** The expression level of EM genes compared to 5,095 genes where the median expression in each tissue is greater than 3 in more than half the tissues. The latter group of genes are expressed at higher levels than the EM genes.

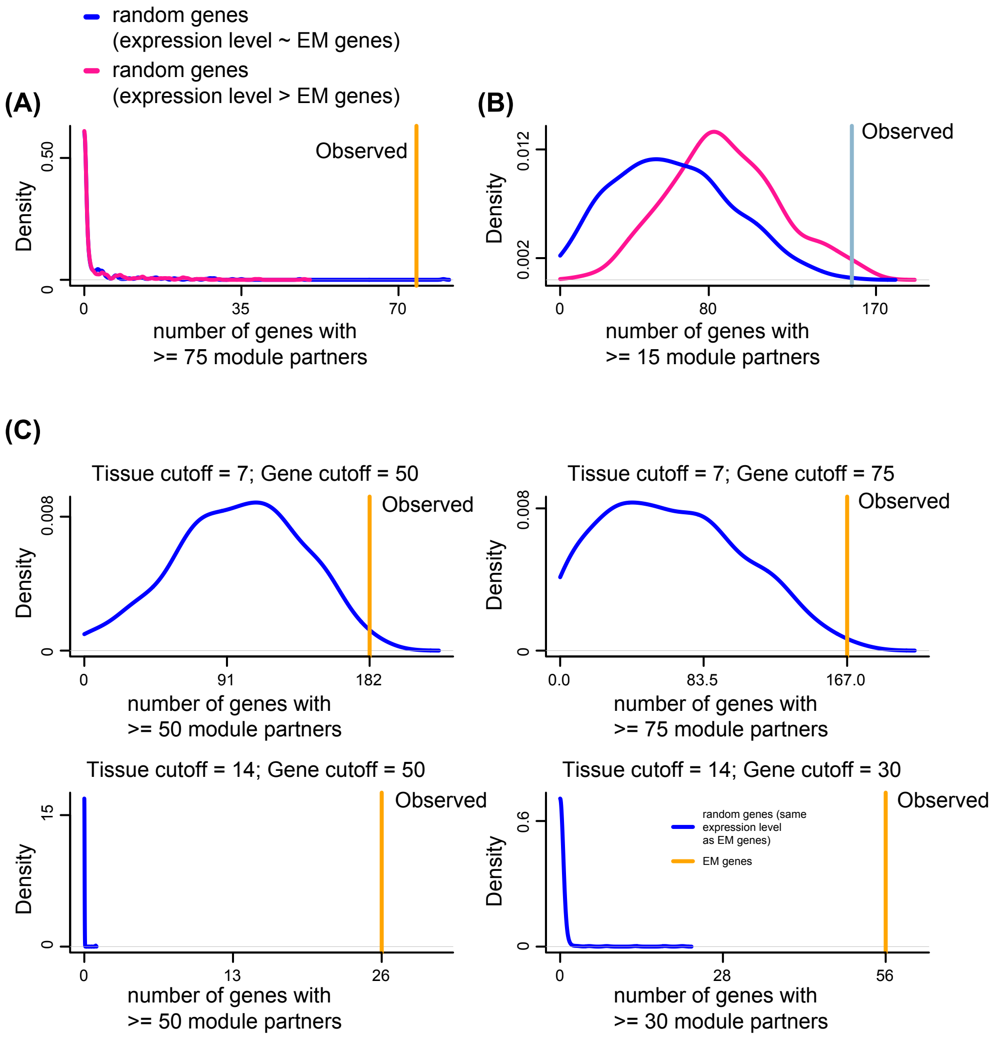

Supplemental Figure S11. EM genes are highly co-expressed irrespective of arbitrary choices. We examine the sensitivity wrt. various cutoffs of the result that EM genes are highly co-expressed. **(A)** Like Figure 4D, but with an additional reference distribution where random genes are selected to have higher expression level than EM genes (Supplemental Fig. S10). **(B)** Like Figure 4D but where we consider our observation that we have 157 EM genes which are either highly co-expressed or co-expressed. **(C)** Like Figure 4D, but for various choices of arbitrary cutoffs. Specifically we vary (1) in how many tissues two genes need to belong to the same module, to be considered partners ("tissue cutoff", in the main text we use 10) and (2) how many module partners a gene needs to have, to be considered part of the highly co-expressed group ("gene cutoff", in the main text we use 75).

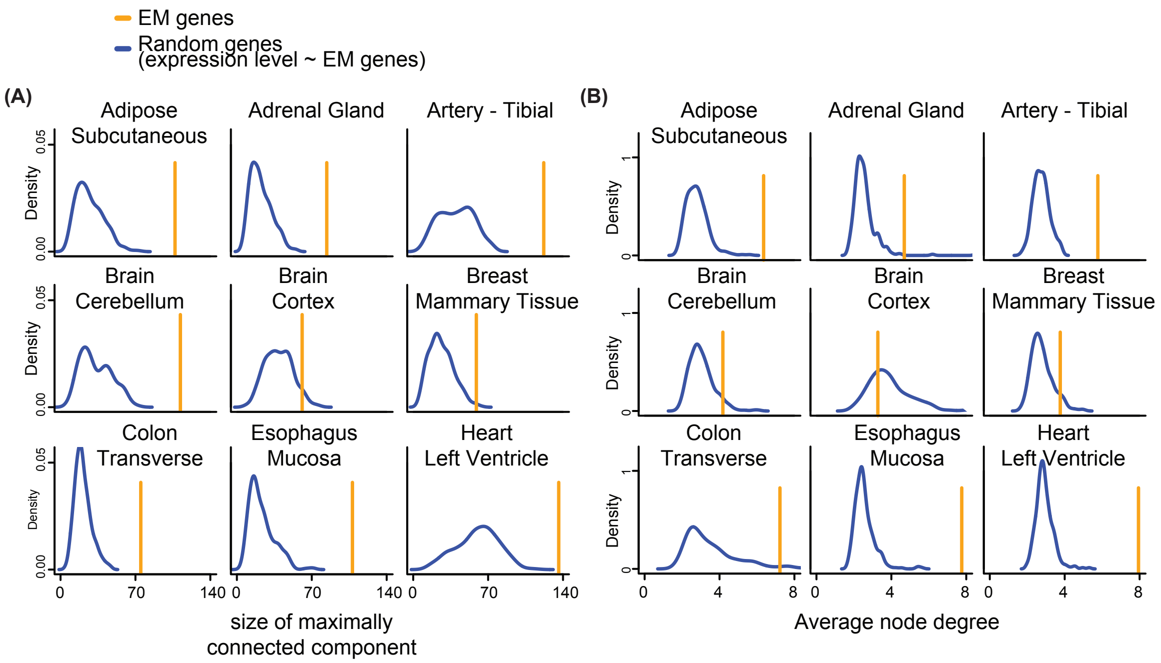

Supplemental Figure S12. Size and average degree of the maximally connected component in different tissues. We estimated tissue-specific networks by thresholding the correlation matrix. For each tissue we computed the maximally connected component of the EM genes. As comparison we did the same for 300 random samples of 270 genes with a similar expression level to the EM genes. Depicted are results from 9/28 tissues (same tissues as in Supplemental Fig. S19). **(A)** The size of the maximally connected component of the EM genes compared to the random samples. **(B)** The average node degree within the maximally connected components.

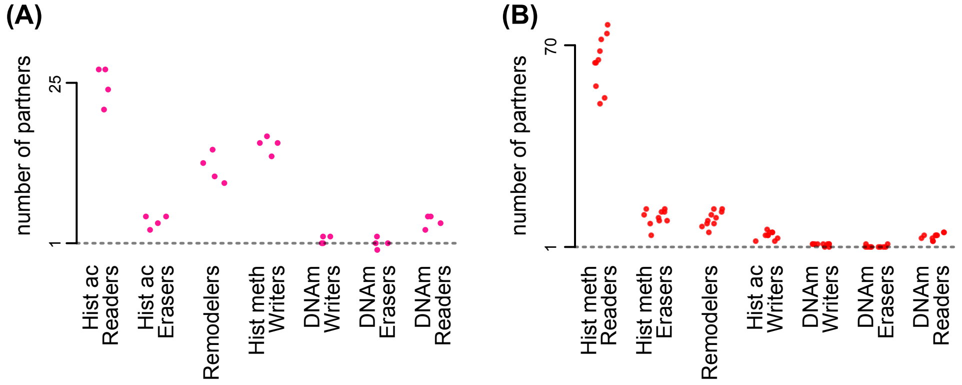

Supplemental Figure S13. Dual function EM writers partner with multiple other EM categories. (A) Each point corresponds to a dual function histone methyltransferase, and the y axis depicts the number of its partners belonging to a given EM category (different positions on the x axis correspond to different categories). **(B)** Same as (A), but for dual function histone acetyltransferases.

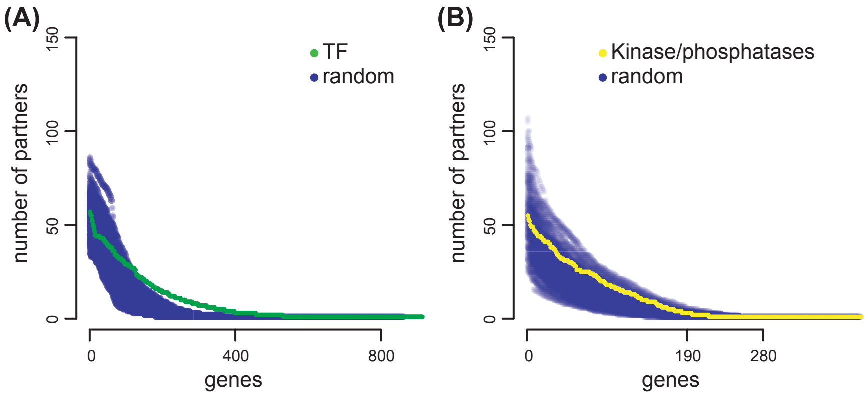

Supplemental Figure S14. Transcription Factors (TFs) and Protein Kinases/Phosphatases are not significantly co-expressed. (A) Each green dot corresponds to a TF, and its position along the y axis corresponds to the number of other TFs that it partners with. The TFs are ordered on the x axis according to the number of their partners. Blue dots correspond to randomly chosen genes, sampled from genes with a median expression (log(RPKM + 1))) greater than 0.2 and less than 2.8 in at least half the tissues, to match the expression of TFs. Each random set contained 915 genes. **(B)** As (A), but for protein kinases/phosphatases (yellow dots). Each random set of genes contained 395 genes, sampled from genes with median expression (log(RPKM + 1))) greater than 0.1 in at least half of the tissues.

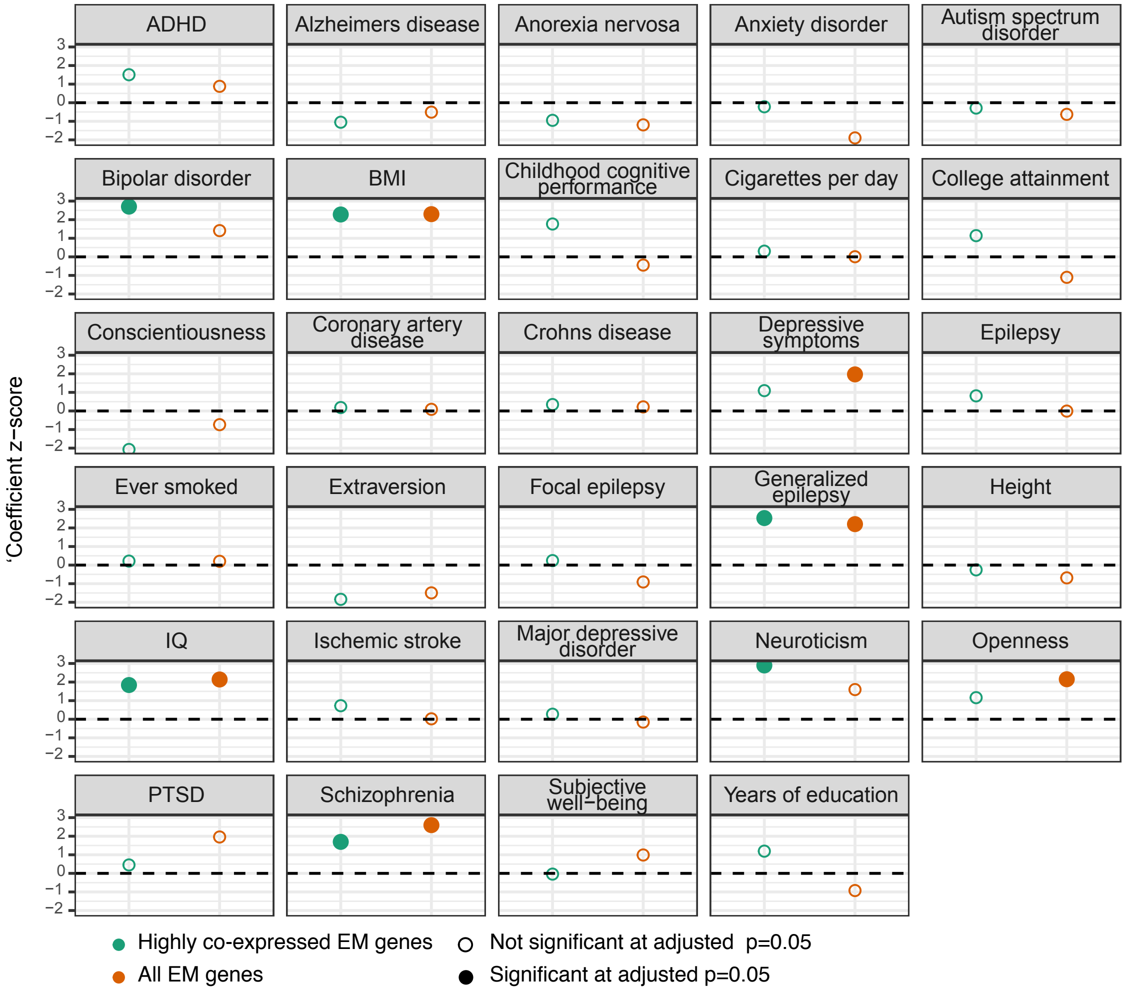

Supplemental Figure S15. Regulatory regions of EM genes are enriched for explained variation for some neurological traits: significance. We performed an LDSC analysis for each of the traits listed in the figure. For each trait we included two different sets of features: regulatory regions for all EM genes (orange) and regulatory regions only for highly co-expressed EM genes (green). As baseline features we included the standard set of LDSC features including conserved regions and coding regions (Methods). For each feature and each trait we computed a coefficient z-score which is a test statistic for whether the feature is significantly enriched for heritability associated with the trait. Filled circles are trait-feature combinations which are significant at 5% after correcting for multiple testing across all feature-trait combinations.

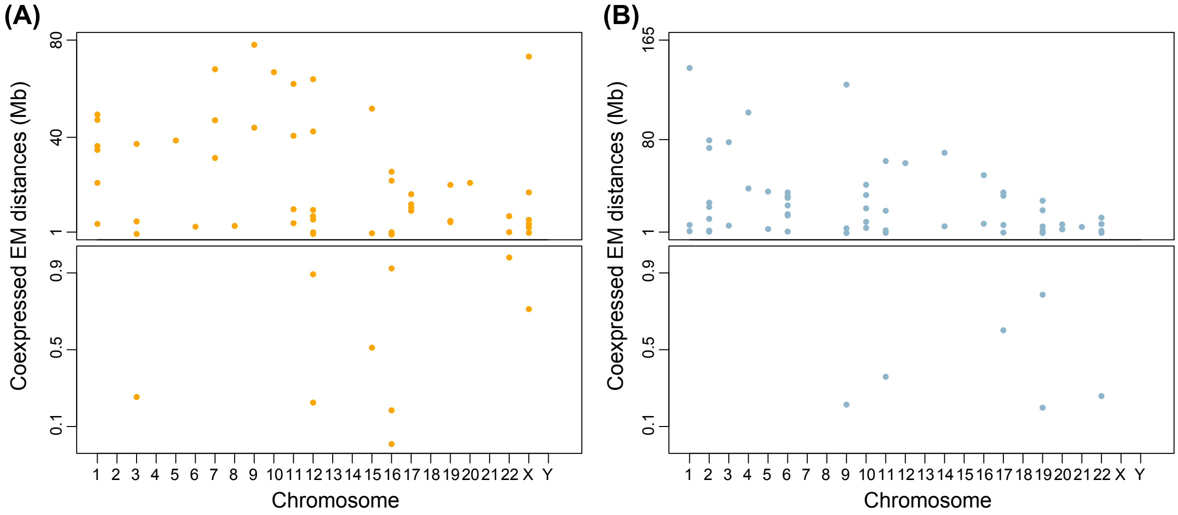

Supplemental Figure S16. Co-expressed EM genes are not spatially clustered. The pairwise chromosomal distances (number of bp separating the transcription end site of a gene with the transcription start site of the most proximal downstream gene) between EM genes. Top panel are distances greater than 1 Mb and bottom panel less than 1 Mb. **(A)** Highly co-expressed EM genes ($n = 74$). **(B)** Co-expressed EM genes ($n = 83$). Both groups of genes are approximately uniformly distributed across chromosomes (chi-squared goodness of fit test, $p = 1$ based on simulation for both groups).

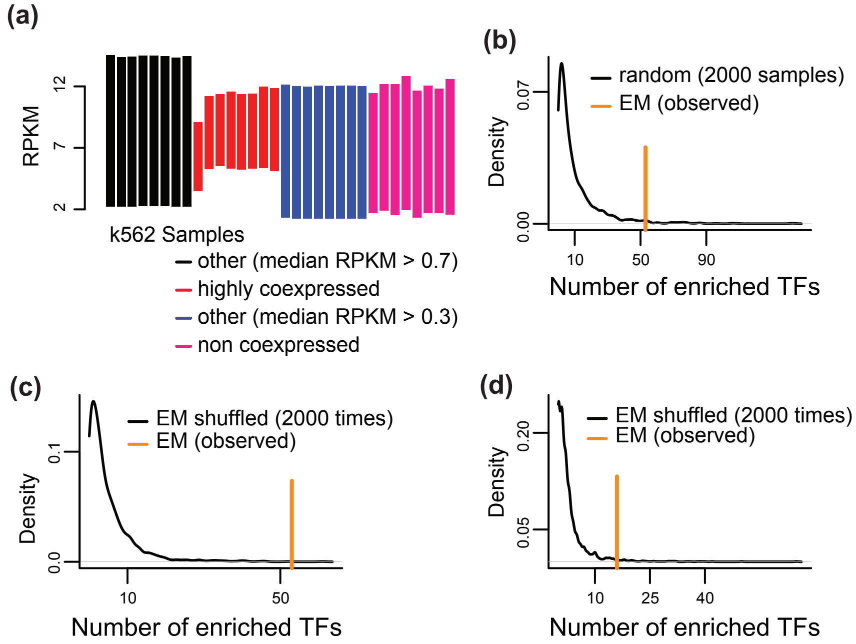

Supplemental Figure S17. The promoters of the highly co-expressed EM genes are bound by common regulatory factors. (A) The distributions of expression levels in K562 cells for genes that were used to derive the reference distribution in **(B)**. **(B)** The distribution of the number of regulatory factors that show enriched binding ($\log_2$ OR $>$ 1 and $p < 0.05$) at the promoters of the first group created after randomly sampling genes in K562 cells (black curve), versus the observed number of regulatory factors showing enriched binding at the promoters of highly co-expressed EM genes (orange vertical line). **(C)** The distribution of the number of regulatory factors that show enriched binding ($\log_2$ OR $>$ 1 and $p < 0.05$) at the promoters of the first group created after shuffling the labels of EM genes in k562 cells (black curve), versus the observed number of regulatory factors showing enriched binding at the promoters of highly co-expressed EM genes (orange vertical line).

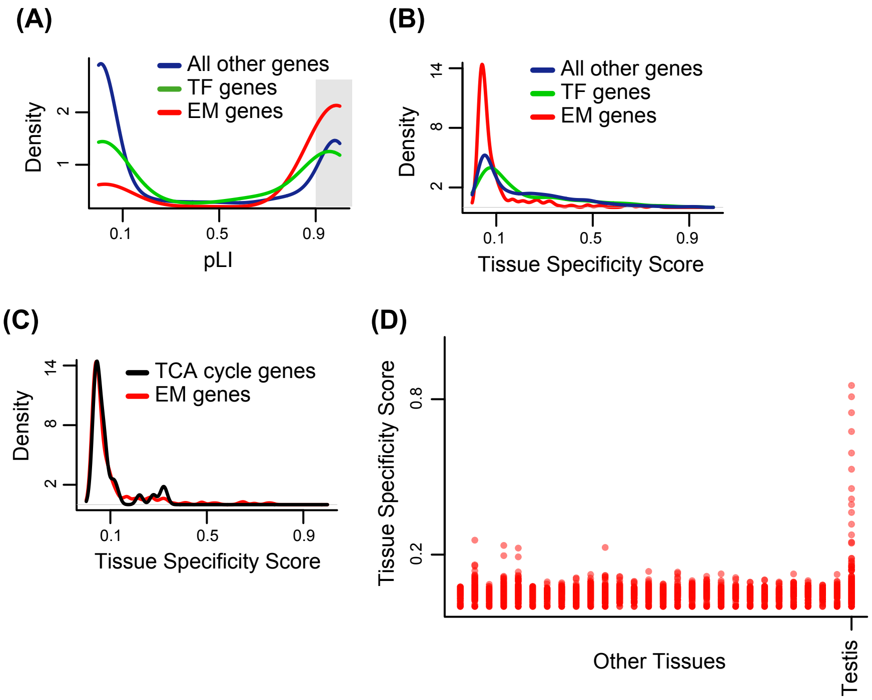

Supplemental Figure S18. The lack of tissue-specificity of EM genes is not driven by unwanted variation. Results for the tissue-specificity analyses after correcting for RIN and surrogate variables (Methods). **(A)** Like Supplemental Fig. S7. **(B)** Like Supplemental Fig. S6A. **(c)** Like Figure S6B. **(D)** Like Supplemental Fig. S6C.

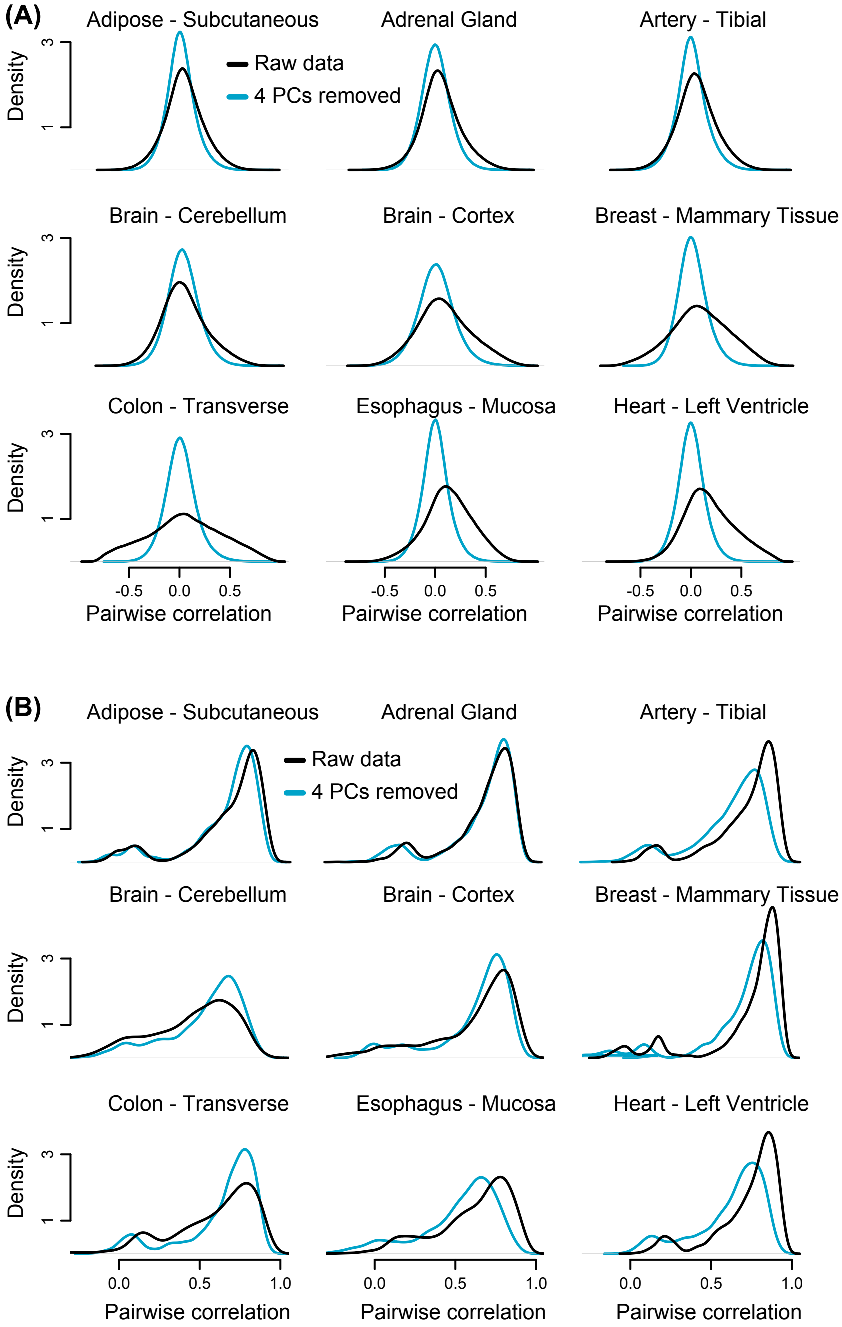

Supplemental Figure S19. Removing noise in co-expression analysis by removing principal components. We remove unwanted variation in our co-expression analysis by removing 4 principal components from the expression matrix in all tissues. **(A)** The distribution of pairwise correlations between randomly sampled genes, serving as a negative control, for 9 out of the 28 tissues. **(B)** The distribution of pairwise correlations between 80 genes coding for ribosomal proteins, which serve as a positive control, for the same tissues as in (A).
